# Cdc42 and k-Ras Control Endothelial Tubulogenesis through Apical Membrane and Cytoskeletal Polarization: Novel Stimulatory Roles for GTPase Effectors, the Small GTPases, Rac2 and Rap1b, and Inhibitory Influence of Arhgap31 and Rasa1

**DOI:** 10.1371/journal.pone.0147758

**Published:** 2016-01-26

**Authors:** Pieter R. Norden, Dae Joong Kim, David M. Barry, Ondine B. Cleaver, George E. Davis

**Affiliations:** 1 Department of Medical Pharmacology and Physiology, University of Missouri School of Medicine, Dalton Cardiovascular Research Center, Columbia, MO, United States of America; 2 Department of Molecular Biology, UT Southwestern Medical Center, Dallas, TX, United States of America; Karolinska Institutet, SWEDEN

## Abstract

A critical and understudied property of endothelial cells is their ability to form lumens and tube networks. Although considerable information has been obtained concerning these issues, including the role of Cdc42 and Rac1 and their effectors such as Pak2, Pak4, Par6b, and co-regulators such as integrins, MT1-MMP and Par3; many key questions remain that are necessary to elucidate molecular and signaling requirements for this fundamental process. In this work, we identify new small GTPase regulators of EC tubulogenesis including k-Ras, Rac2 and Rap1b that act in conjunction with Cdc42 as well as the key downstream effectors, IQGAP1, MRCKβ, beta-Pix, GIT1, and Rasip1 (which can assemble into multiprotein complexes with key regulators including α2β1 integrin and MT1-MMP). In addition, we identify the negative regulators, Arhgap31 (by inactivating Cdc42 and Rac) and Rasa1 (by inactivating k-Ras) and the positive regulator, Arhgap29 (by inactivating RhoA) which play a major functional role during the EC tubulogenic process. Human EC siRNA suppression or mouse knockout of Rasip1 leads to identical phenotypes where ECs form extensive cord networks, but cannot generate lumens or tubes. Essential roles for these molecules during EC tubulogenesis include; i) establishment of asymmetric EC cytoskeletal polarization (subapical distribution of acetylated tubulin and basal membrane distribution of F-actin); and ii) directed membrane trafficking of pinocytic vacuoles or other intracellular vesicles along acetylated tubulin tracks to the developing apical membrane surface. Cdc42 co-localizes subapically with acetylated tubulin, while Rac1 and k-Ras strongly label vacuole/ vesicle membranes which accumulate and fuse together in a polarized, perinuclear manner. We observe polarized apical membrane and subapical accumulation of key GTPases and effectors regulating EC lumen formation including Cdc42, Rac1, Rac2, k-Ras, Rap1b, activated c-Raf and Rasip1 to control EC tube network assembly. Overall, this work defines novel key regulators and their functional roles during human EC tubulogenesis.

## Introduction

In recent years, considerable progress has been made toward our understanding of vascular morphogenesis, including the subject of this manuscript, which addresses how endothelial cells form tube networks with defined lumens [1–6]. Previous work has shown the critical importance of integrins, membrane-type matrix metalloproteinases (MT1-MMP), Rho GTPases, particularly Cdc42 and Rac1, small GTPase regulators such as Rasip1, kinase cascades involving PKCepsilon (PKCɛ), Src family members, Pak2, Pak4, Raf, Mek and Erk, and both the actin and microtubule cytoskeletons [3–5, 7–13]. Other interesting EC lumen regulators are proteins such as the cerebral cavernous malformation (CCM) proteins, CCM1, CCM2, CCM2L, and CCM3, as well as the polarity proteins, Par6b, Par3 and junctional adhesion receptors with affinity for Par3 including JamB, JamC and VE-cadherin [4, 8, 14–19]. An important future direction of this work is to further understand how ECs become polarized during lumen formation [20]. Another critical issue is how defined growth factors work in conjunction with the extracellular matrix to direct EC tubulogenic signaling (through the above key molecular regulators). Recently, we have described that five growth factors together are able to stimulate human EC tubulogenesis in 3D collagen or fibrin matrices under serum-free defined conditions and they are; stem cell factor (SCF), interleukin-3 (IL-3), stromal-derived factor-1α (SDF-1α), fibroblast growth factor-2 (FGF-2) and insulin [21, 22]. How signaling through this combination of growth factors and activated receptors leads to EC lumen and tube formation is a critical and fundamental question that remains to be answered.

The role of polarity regulators has been demonstrated during EC lumen formation (i.e. Cdc42, Par6b, Par3) [8, 14], but how this contributes to the development of an EC apical membrane surface and polarized cytoskeletal apparatus is not well understood. In particular, which membrane trafficking events are necessary to develop the specialized EC apical membrane surface of EC tubes during their formation and following tube maturation events including mural cell recruitment and the exposure of ECs to flow forces? Many years ago, we and others demonstrated that intracellular vacuoles/ vesicles appear to be necessary for the rapid lumen formation ability of ECs when they are exposed to a 3D matrix environment [7, 23–27]. Furthermore, we showed that Cdc42 and Rac1 were necessary for the ability of ECs to form intracellular vacuoles and subsequent lumens [7]. Also, we initially demonstrated that the majority of the vacuoles observed were pinocytic in nature and that both the actin and microtubule cytoskeletons were necessary for their formation [23]. In addition, we showed that other intracellular compartments in ECs, namely Weibel-Palade bodies, were observed to fuse with vacuoles during their transit to the apical domain in that a large proportion of vacuoles contained high amounts of von Willebrand Factor [23]. Our laboratory has now identified many regulators of EC lumen and tube assembly as mentioned above [3, 4, 28], but how these molecules control such critical events including vacuole formation, vacuole fusion with polarized targeting of vesicles to the developing apical membrane, how the cytoskeleton is modified and polarized to specifically direct vacuoles/ vesicles to the apical surface and then how this process is coordinated with MT1-MMP-dependent proteolysis to create EC tube networks that are prepared for the polarized recruitment of pericytes to the abluminal tube surface remains largely unanswered. Thus, there are many fundamental cell biological questions that still need to be investigated with regard to how EC form tube structures during vascular morphogenesis.

We recently demonstrated that EC lumen formation in 3D matrices results in part due to the establishment of asymmetric cytoskeletal polarization with F-actin expressed in a basal fashion and with modified tubulins including acetylated and detyrosinated tubulin localized in a subapical domain to support the developing apical membrane surface [29]. Key plus-end microtubule regulatory proteins, EB1, p150glued and Clasp1, control EC lumen formation through the subapical polarization and expression levels of these modified tubulins [29]. In part they act together to negatively regulate the tubulin deacetylases, HDAC6 and Sirt2. siRNA suppression of these deacetylases singly or in combination, led to increased EC lumen formation, while increased expression of HDAC6 and Sirt2 interfered with lumen formation [29]. In addition, disruption of microtubules with colchicine or other agents such as the chemotherapeutic drug, vinblastine, caused EC tube disassembly and collapse and importantly, there is also rapid loss of tubulin acetylation and activation of RhoA. Thus, tubulin modifications are major regulators of EC lumen formation, but also lumen and tube maintenance via support of the apical membrane domain [29, 30]. A key point is that regulation of the actin cytoskeleton is also crucial for EC lumen formation, in that actin rearrangements downstream of integrin-, Cdc42- and Rac-dependent signaling are necessary to form pinocytic intracellular vacuoles leading to lumen and tube formation [3, 7, 23, 26, 27]. Chemical disruption of either actin or tubulin polymerization (when added from the beginning of the assay) completely blocks EC tubulogenesis [23]. Polarized F-actin at the basal EC surface during lumen formation [29] and EC-EC junctions once tubes have begun to assemble and stabilize is also critical to this process [12]. Thus, the mechanisms that underlie how ECs form tube networks critically involve the EC actin and microtubule cytoskeletons which need to be intricately coordinated during each stage of the process.

Here, in this new study, we have characterized the novel stimulatory role of small GTPases, key downstream effectors, and negative regulators including GTPase activating proteins (GAPs) during EC lumen and tube formation. Our studies have identified important new roles for Cdc42, Rac2, k-Ras and Rap1b and siRNA suppression of Cdc42 in combination with these other three GTPases causes profound inhibition of EC tubulogenesis. Furthermore, we have identified Arhgap31 and Rasa1 as GAPs that interfere with Cdc42, Rac, and k-Ras, and which markedly block the lumen formation process. In contrast, Arhgap29, a Rho-specific GAP, does the opposite and actually stimulates EC tube assembly through its RhoA-inhibitory activity. Additionally, we identify a novel role for downstream effectors of these GTPases including IQGAP1, MRCKβ, beta-Pix, GIT1, and Rasip1 during this process. Finally, we investigate EC polarization during lumen formation and demonstrate the apical targeting of small GTPases through membrane trafficking events along acetylated tubulin-enriched microtubule tracks, as well as the apical membrane targeting of key downstream regulators including Rasip1 and c-Raf.

## Materials and Methods

### Reagents

Stem cell factor (SCF), stromal cell-derived factor 1 alpha (SDF-1α), and interleukin-3 (IL-3) were obtained from R&D systems (Minneapolis, MN). Tubacin was obtained from TOCRIS Bioscience (Bristol, United Kingdom). Ascorbic acid, 12-*O*-tetradecanoyl-phorbol-13-acetate (TPA), and antibodies against Arhgap31, acetylated tubulin, α-tubulin, and phospho-C-Raf Tyr341 were obtained from Sigma-Aldrich (St. Louis, MO). Recombinant fibroblast growth factor 2 (FGF-2) and antibodies against Rac2, detyrosinated tubulin, and β-actin was obtained from EMD Millipore. Antibodies against MRCKβ, k-Ras, Rasip1, GIT1, and MT1-MMP were obtained from Abcam (Cambridge, MA). Antibodies against IQGAP1, ROCK1, and Integrin α2 were obtained from BD Biosciences (San Jose, CA). Antibodies against Rap1B, alpha-Pix, beta-Pix, Cdc42, phospho-p44/42 MAPK (ERK 1/2) Thr202/Tyr204, ERK 1/2, phospho-PAK2 Ser141, PAK2, phospho-PAK4 Ser474, PAK4, phospho-B-Raf Thr401, phospho-B-Raf Ser445, B-Raf, phospho-C-Raf Ser388, C-Raf, phospho-Src Y416, Src, phospho-p38 MAPK Thr180/Tyr182, p38 MAPK, and phospho-Tyr were obtained from Cell Signaling Technologies (Danvers, MA). An antibody against Rasa1 was obtained from Epitomics (Burlingame, CA). Antibodies against RhoA and Rac1 were obtained from Cytoskeleton (Denver, CO). An antibody against Arhgap29 was from Bethyl Laboratories (Montgomery, TX). An antibody against GAPDH was purchased from Research Diagnostics Inc (Flanders, NJ). Alexa fluor^®^ 488 and Alexa fluor^®^ 633 antibodies, and Alexa fluor^®^ 488 and 633 phalloidin were from Molecular Probes (Eugene, OR). WT PKCɛ adenovirus was purchased from Seven Hills Bioreagents (Cincinnati, OH) and WT CSK and DN CSK adenoviruses were purchased from Cell Biolabs (San Diego, CA). GFP, GFP-Cdc42, GFP-Rac1, GFP-RhoA, GFP-N17Rac1, GFP-N19RhoA, GFP-N17Cdc42 and GFP-V12Rac1 adenoviruses were generated as previously described, as well as DN-Pak4 adenovirus, and MT1-ΔC WT and MT1-ΔC EA adenoviruses.

### Vasculogenic tube assembly assays

Human umbilical vein endothelial cells were obtained from Lonza (Walkersville, MD) and were cultured (passage 3–6) as described previously [31]. ECs were then suspended at 2 x 10^6^ cells/mL in 2.5 mg/mL collagen type I matrices and assays were performed as previously described [21, 32]. Briefly, SCF, IL-3, SDF-1α, and FGF-2 were added at 200 ng/mL into collagen type I. Cultures were fed with media containing reduced serum supplement (RSII), ascorbic acid, and FGF-2 at 40 ng/mL. Cultures were allowed to assemble into capillary networks over a period of 0–120 hr when cultures were fixed or collected for further processing. Samples were fixed in 2% paraformaldehyde or 3% glutaraldehyde in PBS. Cultures fixed in paraformaldehyde were then stained for fluorescent microscopy imaging, whereas cultures fixed in glutaraldehyde were stained in 0.1% toluidine blue in 30% methanol. Additionally unfixed collagen gels were lysed to examine protein expression at the indicated time points using standard western blotting techniques. Recombinant adenovirus infection of ECs was performed as previously described [31].

### EC siRNA suppression

siRNA suppression protocols using the siRNA list below were performed as previously described [31]. The cells were allowed to recover for 48 hr and transfection was repeated. The cells were then allowed to recover overnight before being harvested for use in 3D assays.

siRNAs from Ambion are as follows:

Control (AM4637) Silencer Select Negative Control #2

Cdc42 (s2765) 5’-UGGUGCUGUUGGUAAAACA-3’

Rac1 (s11711) 5’-CUACUGUCUUUGACAAUUA-3’

Rac2 (s11714) 5’-CCUCUUUUGGAACAACAUA-3’

RhoA (s758) 5’-CACAGUGUUUGAGAACUAU-3’

k-Ras (s7939) 5’-CUAUGGUCCUAGUAGGAAA-3’

Rap1b (s224515) 5’-AGAUUCUUCGAGUUAAAGA-3’

Pak2 (s10024) 5’-CAGAGGUGGUUACACGGAA-3’

Rasip1 (s29763) 5’-CGAGCUGUUCAAAUCCGAA-3’

Alpha-Pix (s16948) 5’-GUAAAAGCCCUAAAACGAU-3’

Beta-Pix (s18122) 5’-CAACGACAGGAAUGACAAU-3’

GIT1 (s26306) 5’-CCUUGAUCAUCGACAUUCU-3’

Rock1 (s12097) 5’-GGUUAGAACAAGAGGUAAA-3’

Arhgap31 (s33202) 5’-GGACAGAUCUCUACAUAGA-3’

Rasa1 (s11820) 5’-CAUAGAUCACUAUCGAAAA-3’

Arhgap29 (s485) 5’-GACCAAGGCUAAAACGAAU-3’

Stealth siRNAs from Invitrogen are as follows:

Pak4 (NM_001014834_stealth_749) 5’-UGCUUGCGCAGGUCCAUCUUCUUGA-3’

IQGAP1 (NM_003870_stealth_421) 5’-GCCUCCACUUUAGACACACUGAUAA-3’

MRCKβ (NM_006035_stealth 691) 5’-UAAAUCACCACCCACAUAGUAAUCC-3’

### Generation of S-epitope tagged Cherry, Cherry-fusion proteins, and AcGFP-Rasip1 adenoviruses

Cdc42, Rac1, Rac2, RhoA, k-Ras, and Rap1b were amplified from cDNA obtained from Missouri S&T cDNA Resource Center (Rolla, MO) and human Rasip1 and PKCɛ were amplified from cDNA obtained from Open Biosystems (Open Biosystems, GE Dharmacon, Lafayette, CO), and standard restriction digest cloning for individual GTPases and PKCɛ into pmCherry-C1 plasmid and Rasip1 into pAcGFP-C1 (Clontech, Mountain View, CA) using *EcoRI-HF* and *BamHI-HF*, *XhoI* and *BamHI-HF*, and *EcoRI-HF* and *XbaI* restriction enzymes respectively (New England Biolabs). Amplified S-Cherry, S-Ch-GTPase, S-Ch-PKCɛ, and AcGFP-Rasip1 constructs were subcloned into pShuttle-CMV expression plasmid using *NotI-HF*, *XbaI*, *XhoI*, *KpnI-HF* and *SalI-HF* restriction enzymes, respectively (New England Biolabs). Recombinant adenoviral vectors were then generated [33] and propagated as previously described [7]. The PCR primers used are listed below with the upstream first followed by the downstream primer.

Cdc42 5’AGGAATTCTATGCAGACAATTAAGTGTGTTG-3’

5’-AGGGATCCTTAGAATATACAGCACTTCCTTTT-3’

Rac1 5’-AGGAATTCTATGCAGGCCATCAAGTGTGTGGTG-3’

5’-AGGGATCCTTACAACAGCAGGCATTTTCTCTTC-3’

Rac2 5’-AGGAATTCTATGCAGGCCATCAAGTGTGTGGTG-3’

5’AGGGATCCCTAGAGGAGGCTGCAGGCGCGCTTC-3’

RhoA 5’-AGGAATTCTATGGCTGCCATCCGGAAGAAACTG-3’

5’-AGGGATCCTCACAAGACAAGGCACCCAG-3’

k-Ras 5’-AGCTCGAGCTATGACTGAATATAAACTTGTGGTAG-3’

5’-AGGGATCCTTACATAATTACACACTTTGTCTTTG-3’

Rap1b 5’-AGGAATTCTATGCGTGAGTATAAGCTAGTCG-3’

5’-AGGGATCCTTAAAGCAGCTGACATGATGAC-3’

Rasip1 5’-AGGAATTCTATGCTGTCTGGTGAACGGAAGGAGG-3’

5’-AGGTCGACTCAAGGAGACGTGGCCACGGGAGGCCCATG-3’

PKCɛ 5’- AGCTCGAGCTATGGTAGTGTTCAATGGCCTTC-3'

5'-AGGGATCCTCAGGGCATCAGGTCTTCACCAAAG-3'

Primers used for cloning into the pAdCMVShuttle plasmid are as follows. The first two primers (S-Cherry and AcGFP) are upstream primers while the following ones are downstream primers.

S-Cherry 5’-AGGCGGCCGCACCATGGCAAAAGAAACCGCTGCTGCGAAATTTGAACGCCAGCACATGGACTCGATGGTGAGCAAGGGCGAGGAG-3’

AcGFP 5’-AGGGTACCACCATGGTGAGCAAGGGCGCCGAGCTGTTCAC-3’

Cdc42 5’-AGTCTAGATTAGAATATACAGCACTTCCTTTT-3’

Rac1 5’-AGTCTAGATTACAACAGCAGGCATTTTCTCTTC -3’

Rac2 5’-AGTCTAGACTAGAGGAGGCTGCAGGCGCGCTTC-3’

RhoA 5’-AGTCTAGATCACAAGACAAGGCACCCAG-3’

k-Ras 5’-AGCTCGAGTTACATAATTACACACTTTGTCTTTG-3’

Rap1b 5’-AGTCTAGATTAAAGCAGCTGACATGATGAC-3’

PKCɛ 5’-AGTCTAGATCAGGGCATCAGGTCTTCACCAAAG-3'

Rasip1 5’-AGGTCGACTCAAGGAGACGTGGCCACGGGAGGCCCATG-3’

### EC vasculogenesis pull-down assay

Pull down assays using S-epitope tagged mCherry GTPase fusion protein expressing adenoviruses were performed similarly to that previously described [31]. EC vasculogenesis assays were set up in 3.75 mg/mL collagen type I gels using adenovirus infected ECs and extracted at the indicated time points. 3D cultures were placed in lysis buffer consisting of 1% Triton X-100, 10 mM Tris-base (pH 7.5), 150 mM NaCl, 1 mM DTT and 5 mM MgCl_2_, or 1 mM MgCl_2_ and 1 mM CaCl_2_, Complete EDTA-free protease inhibitor cocktail tablets (Roche Diagnostics, Indianapolis, IN), collagenase (150 μg/μL high-purity; Sigma-Aldrich), and 100 μM GTPγS (Calbiochem). Lysates were incubated in a 37°C water bath for 15 minutes to aid in collagen digestion and were clarified by centrifugation at 16,000g for 20 minutes at 4°C. Supernatants were then incubated with S-protein agarose beads (Novagen, EMD Millipore, Billerica, MA) equilibrated with washing buffer (respective lysis buffer containing 0.1% Triton X-100) for 1 hour at 4°C on a rocking plate. Beads were then washed 4 times with washing buffer before bound protein was eluted with 1.5X sodium dodecyl sulfate sample buffer containing 7.5% β-Mercaptoethanol. Bound GTPase-associated proteins were detected by western blot analysis.

### In vitro culture immunofluorescent staining, microscopic imaging, and analysis

For analysis of 3D cultures, immunostaining was carried out as previously described. Immunostained cultures were imaged using a confocal microscope (Leica TC5 SP5) connected to a multiphoton system (Leica, Buffalo Grove, IL) using excitation wavelengths of 488 nm and 543 nm or 488 nm and 633 nm sequentially. High-resolution images were captured using a 63X water immersion objective (NA 1.2) utilizing Leica Application Suite (LAS) software. Toluidine blue stained cultures were imaged using light microscopy on inverted microscopes (Eclipse TE2000-E; Nikon, Melville, NY with Photometrics CoolSNAPHQ2 camera, Tucson, AZ, and Olympus CKX41 with Olympus DP70 camera, Center Valley, PA). Photographs were analyzed using Metamorph software (Molecular Devices, Sunnyvale, CA) by tracing vessel area and lumen area. Time-lapse video microscopy was performed using light microscopy and a 20X objective with a fluorescent inverted microscope (DMI6000B, Leica) over a 72 hr period.

### Immunofluorescent and immunocytochemical staining of embryonic tissues

Female mice expressing the Rosa26 yellow fluorescent protein (YFP) reporter were mated with male mice expressing Cadherin5-CreERT2 [12, 34]. Pregnant females were induced with tamoxifen (2mg/40g Mouse) at E12 and E13, and then embryos dissected at E14. Head dermis positive for YFP was isolated, fixed in 4% PFA/PBS for 1hr at 4°C then washed in PBS. Primary antibody incubations were carried out at 4°C O/N (diluted 1:300 for GFP, 1:100 for Rasip1), slides were washed in PBS, and then incubated in secondary antibody for 4 hrs at RT (diluted 1:500). Slides were washed in PBS incubated with DAPI and mounted. Images were obtained using a LSM710 Meta Zeiss confocal. Antibodies used include: anti-GFP (Aves/ GFP-1020), Rasip1 (Novus Biologicals/ NB300-967), and Alexa Fluor 555 donkey anti-goat (Invitrogen/ A21432, Donkey anti-chicken 488 (Jackson Immuno Cat#703-545-155). Rasip1^+/-^ and Rasip1^-/-^ embryos were fixed in 4% PFA/PBS and stored in 75% ethanol. Embryos were processed and sectioned as described. Sections were incubated with primary antibody (diluted 1:100 PECAM, 1:100 Endomucin) overnight at 4°C, and after washing, were incubated with Donkey anti-Rat HRP secondary antibody (diluted 1:100). The DAB reaction was performed using a peroxidase substrate kit (Vector). The slides were imaged using a NeoLumar stereomicroscope (Zeiss). Antibodies used include: Goat anti-rat IgG (Santa Cruz/ A10549), PECAM (BD Biociences/ 553370), Endomucin (Santa Cruz/ sc-65495).

### Statistical analysis

Statistical analysis was completed using Microsoft Excel. Statistical significance was set at minimum with P < 0.05. Student t-tests were used when analyzing two groups within individual experiments (with a minimum n = 10).

### Ethics Statement

All animal studies were performed in accordance with UT Southwestern Medical Center Institutional Animal Care and Use Committee (IACUC) approved protocol APN 2008–0310, approval date September 25, 2014. Mice are housed in a modern air-conditioned facility and are maintained according to NIH guidelines. In addition, the facilities are supervised by full time veterinarians and technical staff and are fully accredited by the American Association for Accreditation of Laboratory Animal Care. Mice were rapidly euthanized by CO_2_ gas and asphyxiation which minimizes pain and discomfort and this method is consistent with the recommendations of the AVMA Panel of Euthanasia.

## Results

### Identification of new small GTPase regulators of EC tubulogenesis

**Fig 1:**
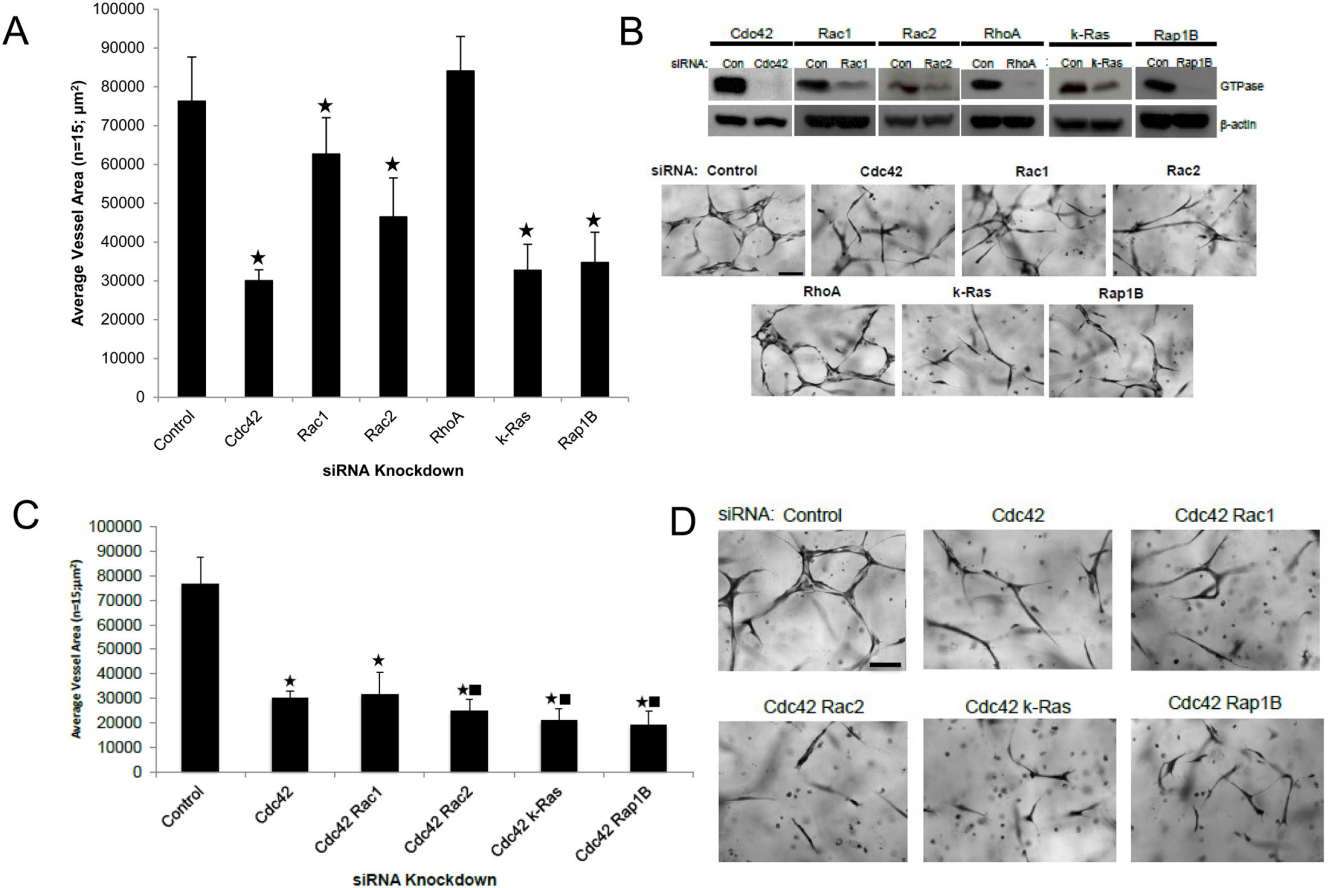
Identification of key small GTPases that control EC tubulogenesis in 3D matrices. (A) Individual EC cultures were transfected with control siRNA or with siRNAs directed to Cdc42, Rac1, Rac2, RhoA, k-Ras, and Rap1b, and then were suspended in 3D collagen matrices for 72 hr using the Factor-induced model. Data are reported as the mean total vessel area per high-power field (HPF) ± standard deviation (SD) (*n* = 15, p < 0.01). Asterisk indicates significance compared to control cultures. Fixed cultures were fixed, stained and photographed. Bar equals 25 μm (B). (B) Lysates generated from siRNA transfected cultures in (A) were used in Western blots to assess specific protein knockdown versus control. (C,D) Individual EC cultures were transfected with siRNA targeting Cdc42, combinations of Cdc42 with Rac1, Rac2, k-Ras, and Rap1B siRNAs, or a control siRNA. EC tubulogenesis assays were performed with the cells the Factor-induced model and after 72 hr, cultures were fixed, photographed (D) and quantitated (C). Data are reported as the mean vessel area ± SD (n = 15; p < 0.01). Asterisk indicates significance compared to control while the square indicates significance compared to Cdc42 siRNA treatment. Bar equals 25 μm.

In this new study, we performed a broad small GTPase screen using siRNA suppression where we identified several new regulators as well as effectors of these GTPases (Fig 1 and see later on). We demonstrate a key role for k-Ras, Rac2 and Rap1b during this process (Fig 1 and S1 Fig). For comparison, siRNAs directed to Cdc42 and Rac1 also inhibit EC tube formation, while siRNA suppression of RhoA had no influence (Fig 1). We have previously reported similar findings with regard to Cdc42, Rac1 and RhoA [8]. To address these questions using a different experimental strategy, we increased expression of these GTPases using wild-type proteins that were fused on their N-terminus with mCherry and an S-epitope tag (for biochemical pulldown assays). Increasing expression of Cdc42, k-Ras, and Rap1b all significantly enhanced EC lumen formation, while others did not (S1 Fig). Using combinations of siRNAs, we demonstrated that knockdown of Cdc42 with k-Ras, Rac2, and Rap1b, appear to have the greatest blocking influence (Fig 1C and 1D) compared to Cdc42 knockdown alone. Combined knockdown of k-Ras with Rac1, Rac2 or Rap1b did not block in a significant manner compared to k-Ras alone (not shown), suggesting the possibility of signaling overlap downstream of these GTPases. Overall, this work suggests that Cdc42-dependent signaling in combination with either k-Ras, Rac2 and Rap1b, appears to be particularly critical for ECs to form lumens and tubes in a 3D matrix environment.

### Functionally interchangeable Factor- and Phorbol ester-induced human EC lumen and tube formation models

**Fig 2:**
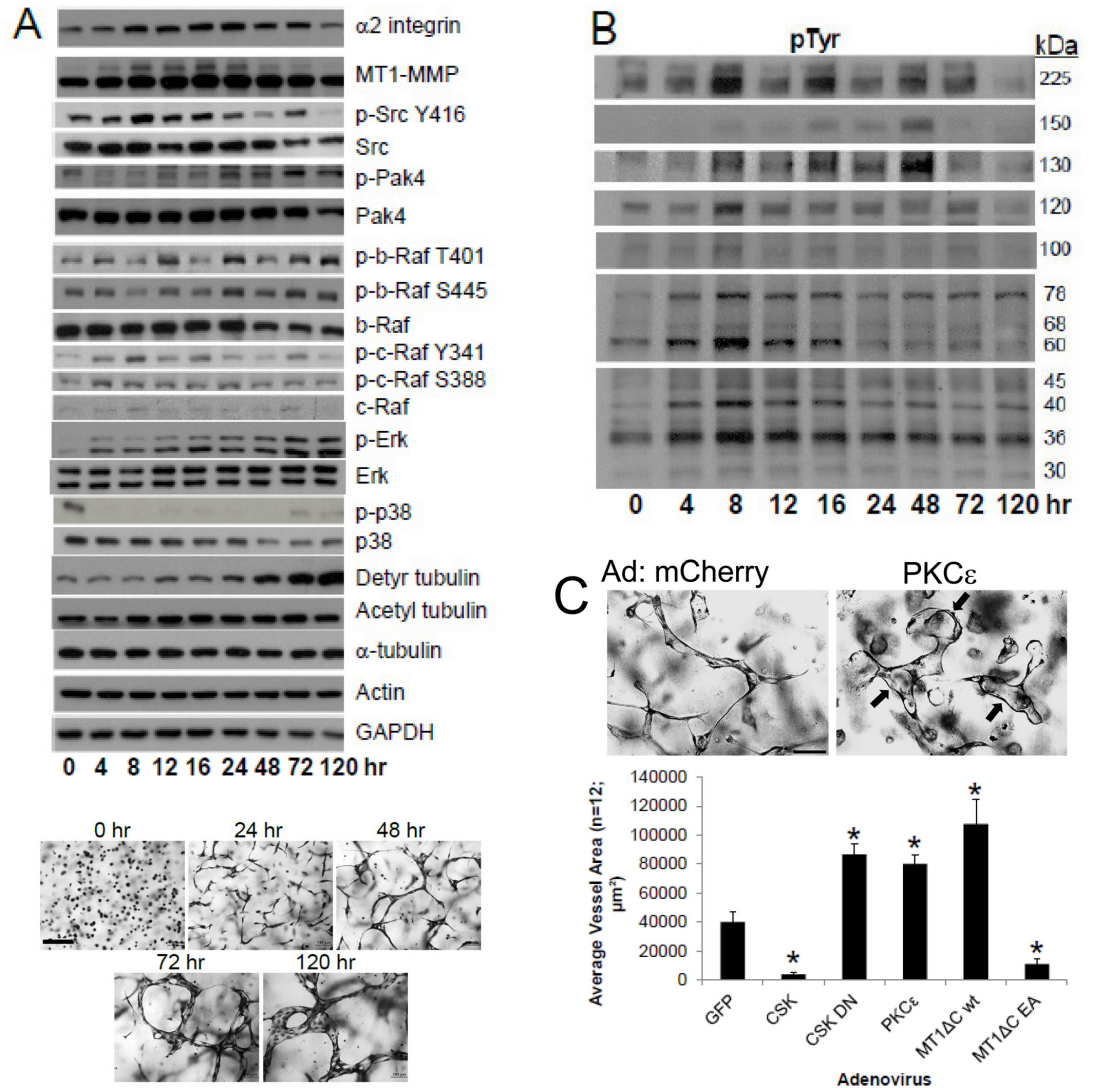
Signaling events that characterize EC tubulogenesis over time in 3D collagen matrices. (A) ECs suspended in 3D collagen matrices using the Factor-induced model were lysed at the indicated time points for Western Blot analysis to assess expression and signaling of the indicated molecules or were fixed and photographed while ECs are assembling into tubes over time. Bar equals 50 μm. (B) The same time courses were analyzed for phosphotyrosine (pTyr)-containing proteins and increased levels of pTyr bands at 225, 150, 130, 120, 100, 76, 68, 60, 45, 40, 36, and 30 kDa were identified over time correlating with the EC tubulogenic process. (C) ECs were infected with the indicated recombinant adenoviruses and then a lumen assay was performed over 72 hr. Upper panel- ECs were infected with mCherry or PKCɛ adenoviruses, then cultured in 3D collagen gels using the Factor-induced model, fixed and photographed. Bar equals 50 μm. Lower panel- ECs were infected with adenoviruses carrying GFP, Csk, DN Csk, PKCɛ, tail-deleted catalytically active WT MT1-MMP (MT1-ΔCT wt) or a dominant negative MT1-MMP construct (MT1-ΔCT EA), then suspended in 3D collagen matrices to assess their ability to form lumens. Data are reported as the mean vessel area ± SD per HPF (n = 12; p < 0.01). Asterisks indicate significance compared to control GFP cultures.

Our studies have utilized two highly defined and related systems which have allowed us to investigate the human EC lumen formation process. One of them utilizes defined growth factors (Factor-induced model) that drive EC tubulogenesis, while the other depends on the addition of phorbol ester [21, 23, 31]. To characterize this new Factor-induced system for comparison with previous studies [9, 29], we have performed detailed Western blots to assess signaling pathways and requirements during the tubulogenic process (Fig 2). Using the Factor-induced model, lumen formation is markedly stimulated by PKCɛ, dominant negative Csk (to activate Src kinases) as well as a cytoplasmic tail deleted wild type MT1-MMP construct (Fig 2C), all of which mimic what we reported in past work [9, 14]. Furthermore, we demonstrate that increased expression of Csk, to block Src activation, addition of the Src inhibitor, PP2 (but not PP3, its inactive control) (not shown) or expression of a dominant negative inhibitor of MT1-MMP (cytoplasmic tail deleted combined with an inactivating mutation) all dramatically interfere with lumen formation just like we previously observed (Fig 2C) [9, 14], suggesting that our two model systems appear to be functionally interchangeable. A final point is that we previously reported that increased tubulin acetylation and detyrosination accompany lumen formation and are necessary to stabilize the developing EC apical membrane using the phorbol ester model [29]. Here, we show the same increases in tubulin acetylation and detyrosination during lumen formation using the Factor system (Fig 2), and furthermore, addition of the HDAC6 inhibitor, tubacin, which strongly increases tubulin acetylation, leads to marked increases in lumen formation (S1 Fig).

### Differential signaling events during Factor-induced EC lumen and tube formation

During the time course of lumen formation using the Factor-induced model, we observe increases in the expression of both α2β1 integrin and MT1-MMP, as well as increased phosphorylation of Src, Pak4, B-Raf, c-Raf, and Erk, while levels of p38 Map kinase phosphorylation remain low compared to controls (Fig 2A). We also performed Western blots over time to assess whether changes in proteins that are tyrosine phosphorylated are differentially regulated during lumen formation. We observe increased tyrosine phosphorylation of bands during this process at 225, 150, 130, 120, 100, 78, 68, 60, 45, 40, 36, and 30 kDa (Fig 2B). EC tubulogenesis is Src family- and receptor tyrosine kinase-dependent through Factor and extracellular matrix signaling events and work is ongoing to identify these tyrosine phosphorylated bands that are induced during this process.

### Small GTPase targeting and polarization to apical membranes and the microtubule cytoskeleton enriched in acetylated tubulin during EC lumen formation

**Fig 3:**
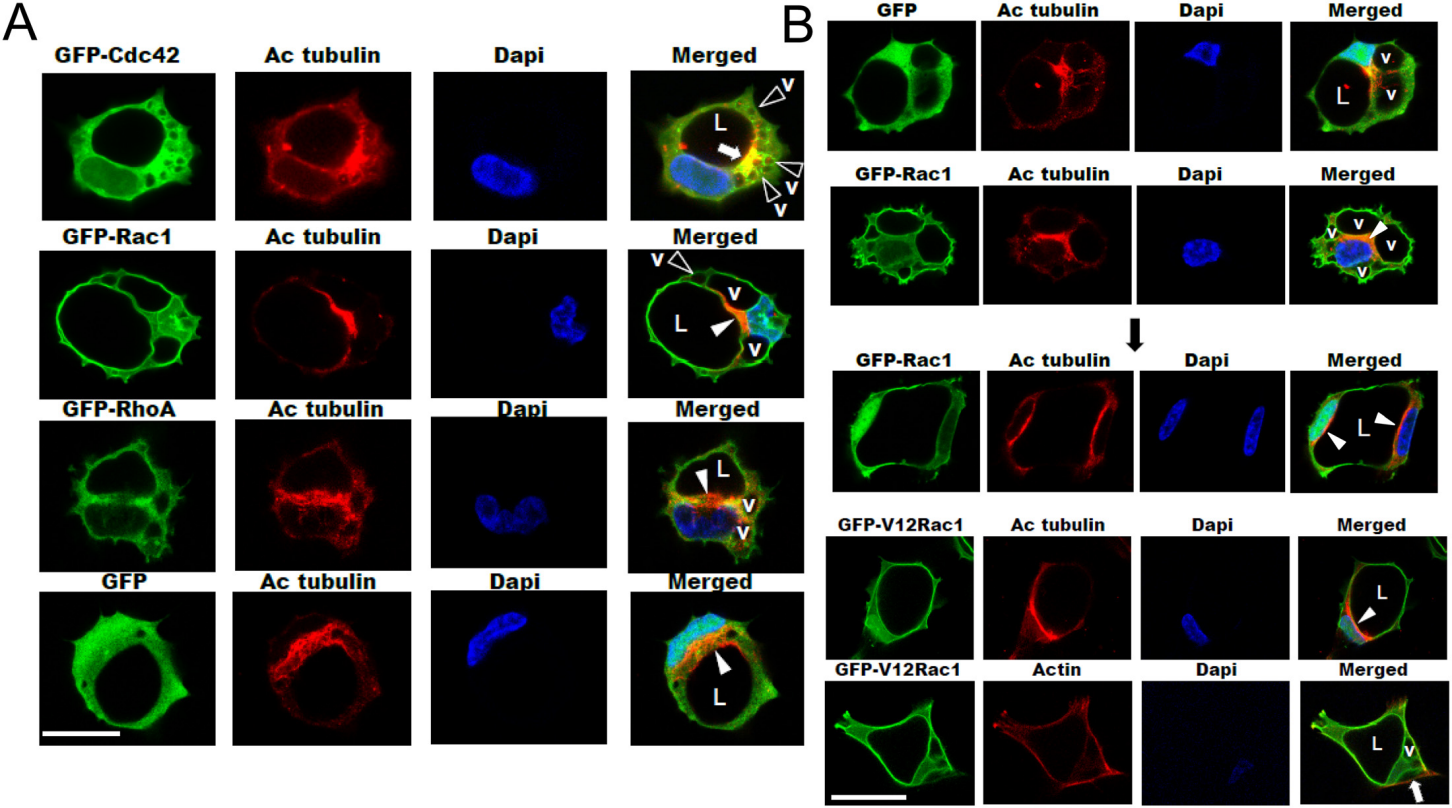
Apical polarization of Cdc42, Rac1 and acetylated tubulin during EC tubulogenesis: Membrane trafficking of vacuoles along microtubule tracks toward the apical surface directs EC lumen formation in 3D matrices. (A) ECs were infected with recombinant adenoviruses carrying GFP-Cdc42, GFP-Rac1, GFP-RhoA, or GFP and were allowed to form vacuoles and lumens in 3D collagen matrices for 12–16 hr to visualize early events in this process using the phorbol ester-induced model. Fixed cultures were then immunostained for acetylated tubulin, analyzed by confocal microscopy, and representative images are shown. White arrowheads indicate subapically polarized acetylated tubulin staining, black arrowheads indicate vacuoles (v). White arrow indicates co-localization of Cdc42 with acetylated tubulin expression in a polarized subapical domain. Vacuoles appear to be in contact with tubulin cytoskeletal tracks that are enriched in acetylated tubulin that coordinate vacuole transport and fusion events at the EC apical membrane surface. L indicates the EC lumen space. Bar equals 25 μm. (B) ECs were infected with recombinant adenoviruses carrying GFP-Rac1, GFP-V12Rac1, or GFP control and after 12 (upper two panels) or 16 hr (lower three panels), cultures were fixed and stained with either anti-acetylated tubulin antibodies or with phalloidin to label F-actin. White arrowheads indicate apical polarization of acetylated tubulin and v indicates vacuoles. L indicates lumen space and the white arrow indicates basal polarization of F-actin. Bar equals 25 μm.

One of the key steps in lumen formation is creation of a unique apical surface. We are putting forth a considerable effort in our laboratory to define how the apical surface forms and stabilizes during EC lumen formation. For example, we recently showed that the modified tubulins, acetylated and detyrosinated tubulin are subapically polarized during lumen formation (Fig 3) and this depends on the microtubule plus-end regulators, EB1, p150glued and Clasp1 [29]. Blockade of these molecules results in interference with lumen formation and apical polarization. By contrast, F-actin as visualized using phalloidin, is polarized in a basal location. Here, we have imaged the subcellular localization of small GTPases (using fluorescent fusion proteins) that directly influence this process. Cdc42, Rac1, Rac2, k-Ras, and Rap1b all show targeting ability to the apical surface in comparison to the basal distribution of F-actin (Fig 3 and S1 Fig). In addition, a key activated effector downstream of these GTPases is phospho-c-Raf [9, 14], which also shows apical targeting during these events (S1 Fig).

Furthermore, we have examined the relationship between Cdc42 and Rac1 and the appearance of these proteins in the apical domain along with acetylated tubulin, which is strongly polarized subapically (Fig 3). Cdc42 concentrates in a subapical distribution that also shows focal co-localization with acetylated tubulin (Fig 3). By contrast, although acetylated tubulin is subapically distributed, GFP (control) or RhoA do not display apical localization. We also performed experiments by expressing constitutively active GFP-V12Rac1 (which enhances lumen formation) [7], which stimulated the appearance of acetylated and detyrosinated tubulin and promoted localization of acetylated tubulin subapically (Fig 3 and S2 Fig). Expression of dominant negative Cdc42, Rac1, and Pak4 constructs (which block lumen formation) [7, 8] reduced acetylated tubulin compared to control or dominant negative RhoA expression (S2 Fig). Using GFP-Rac1, GFP-V12Rac1, GFP-Cdc42 constructs versus control GFP or GFP-RhoA constructs, we are able to observe pinocytic intracellular vacuoles [7, 23] which traffic apically to fuse along modified tubulin cytoskeletal tracks in a subapical and perinuclear region to contribute to the EC apical luminal membrane over time (Fig 3).

### Arhgap31 (a Cdc42 and Rac GAP) and Rasa1 (a Ras GAP), inhibit EC lumen formation, while Arhgap29 (a RhoGAP), stimulates this process

**Fig 4:**
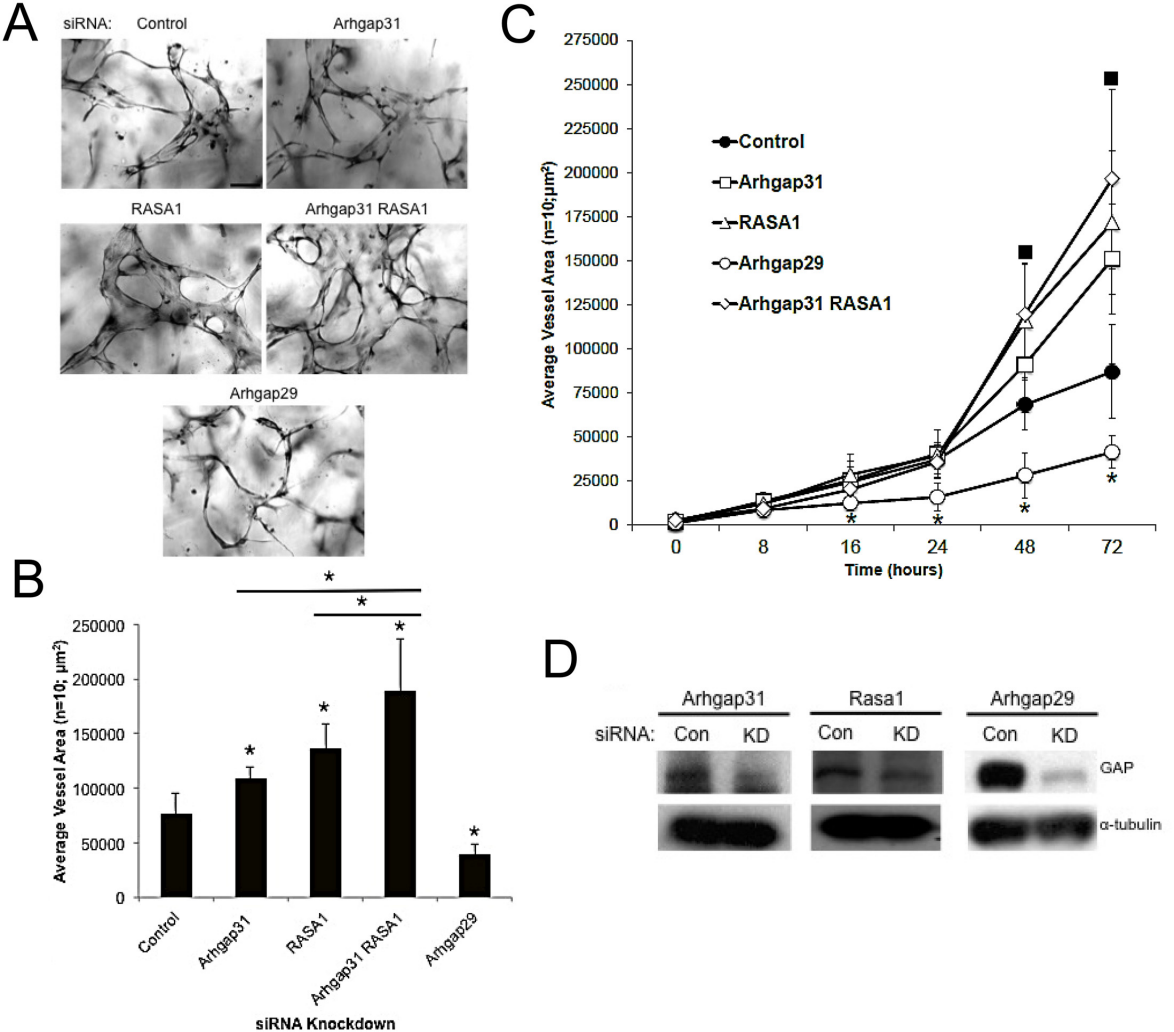
EC tubulogenesis is negatively regulated by the GAPs, Arhgap31 and Rasa1, and is positively regulated by Arhgap29. Individual EC cultures were transfected with a control siRNA or siRNAs directed to Arhgap31, Rasa1, Arhgap29, or the combination of Arhgap31 and Rasa1. Treated ECs were then suspended in 3D collagen matrices for 72 hr using the Factor-induced model, were fixed, photographed and lumen formation was quantitated. (A) Representative images of control, Arhgap31, Rasa1, Arhgap31 and Rasa1, and Arhgap29 siRNA transfected EC 3D cultures are shown. Bar equals 50 μm. (B) Cultures from (A) were quantified for EC tube formation (B). Data are reported as mean vessel area ± SD per HPF (n = 10; p < 0.01). Asterisk indicates significance compared to control. (C) ECs were treated with the indicated control or GAP siRNAs, lumen assays were performed, fixed, and quantitated at the indicated time points. Squares indicate significance at p < .01 above control while asterisks indicate significance below control (n = 10). (D) siRNA transfected EC lysates were examined for the degree of protein knockdown using Western Blot analysis.

To address the role of specific GTPases in EC lumen formation using a distinct approach, we have identified three GTPase activating proteins (GAPs) (which inactivate the GTPases) that control the EC lumen formation process (Fig 4). Arhgap31 shows specificity for Cdc42 and Rac [35], Rasa1 for Ras [36], and Arhgap29 for RhoA [11]. Arhgap29 is a known binding partner of Rasip1, a key regulator of EC lumen formation [11]. siRNA suppression of Arhgap31 and Rasa1 (or in combination) strongly stimulated EC lumen and tube formation, while suppression of Arhgap29 strongly inhibited (Fig 4). This latter result is consistent with previous observations from our laboratory where RhoA played a direct role in vessel collapse following microtubule disruption [30]. Increased expression of activated RhoA completely inhibited EC lumen formation [7] and interestingly, infection of ECs with the microorganism, *Bartonella bacilliformis*, which led to RhoA activation, similarly blocked EC tube formation [37].

**Fig 5:**
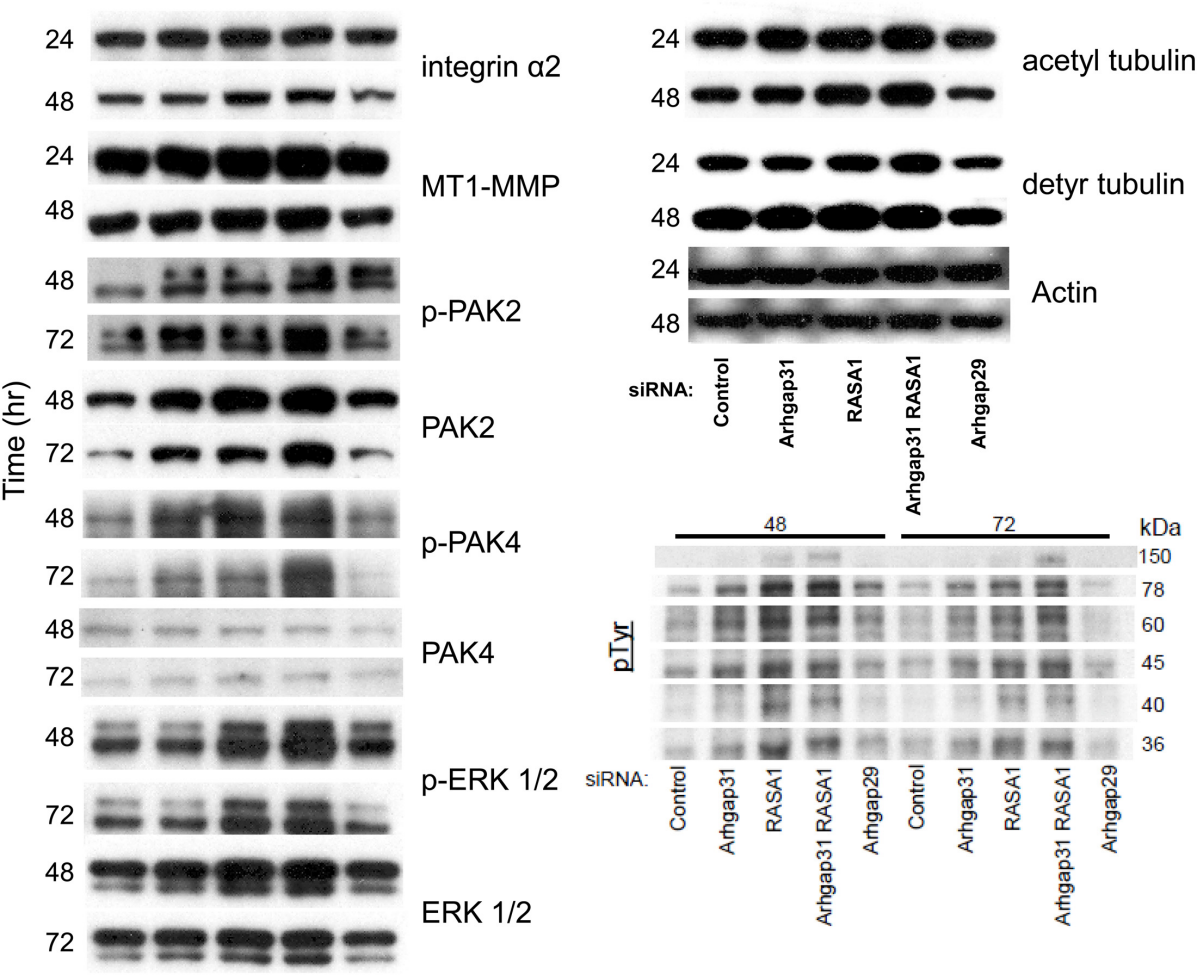
Increased Cdc42, Rac, and Ras activity via siRNA suppression of Arhgap31, Rasa1 or both stimulates key signaling pathways and molecules that control EC tubulogenesis. Individual EC cultures were transfected with control siRNA or siRNA targeted against Arhgap31, Rasa1, Arhgap31 and Rasa1, or Arhgap29 and suspended in 3D collagen gel matrices using the Factor-induced model. Lysates were generated at the indicated time points (24, 48, and 72 hr) from 3D cultures for Western Blot analysis to assess the expression of α2 integrin, MT1-MMP, p-Pak2, Pak2, p-Pak4, Pak4, p-Erk, Erk, acetylated tubulin, detyrosinated tubulin, tyrosine phosphorylated proteins, and actin over time.

To assess the influence of GAP knockdown on EC signaling during lumen formation, we performed Western blots at different time points during the process. This approach allows us to assess the relative contribution of Cdc42/Rac, k-Ras, and RhoA toward the signaling pathways that are activated during lumen formation (Figs 2 and 5). Interestingly, the expression of α2β1 integrin and MT1-MMP were modestly increased when Arhgap31 and Rasa1 are suppressed compared to control, and similar findings were observed with Pak2. Erk1/2 levels modestly increased selectively with Rasa1 knockdown that accompany stronger increases in Erk1/2 phosphorylation. We observe increased phosphorylation of Pak2 and Pak4 that occur following knockdown of either Arhgap31 or Rasa1 (or the combination). Since Pak4 is a selective effector of Cdc42, this result suggests that activation of k-Ras (via Rasa1 knockdown) leads to increased activation of Cdc42, demonstrating that these pathways are co-activating each other (perhaps through upstream guanine exchange factors- GEFs). Similar results were observed with Pak2 activation where Rasa1 knockdown increases Pak2 phosphorylation, an effector of both Cdc42 and Rac1/2. We also examined tyrosine phosphorylation of substrates in these samples and observed that bands at 150, 78, 60, 45, and 36 kDa were increased when either Arhgap31 or Rasa1 (or both) were suppressed, while a band at 40 kDa appeared to be increased more selectively with Rasa1 knockdown. These data suggest that these phosphoproteins may be involved in the lumen formation process since their phosphorylation patterns correlate with EC tube formation ability (Fig 5) and they were also regulated during a time course of tube formation (Fig 2). Future work will attempt to identify these proteins and to determine their functions during these events.

### Identification of new Cdc42, Rac and Ras effectors which control EC tubulogenesis

**Fig 6:**
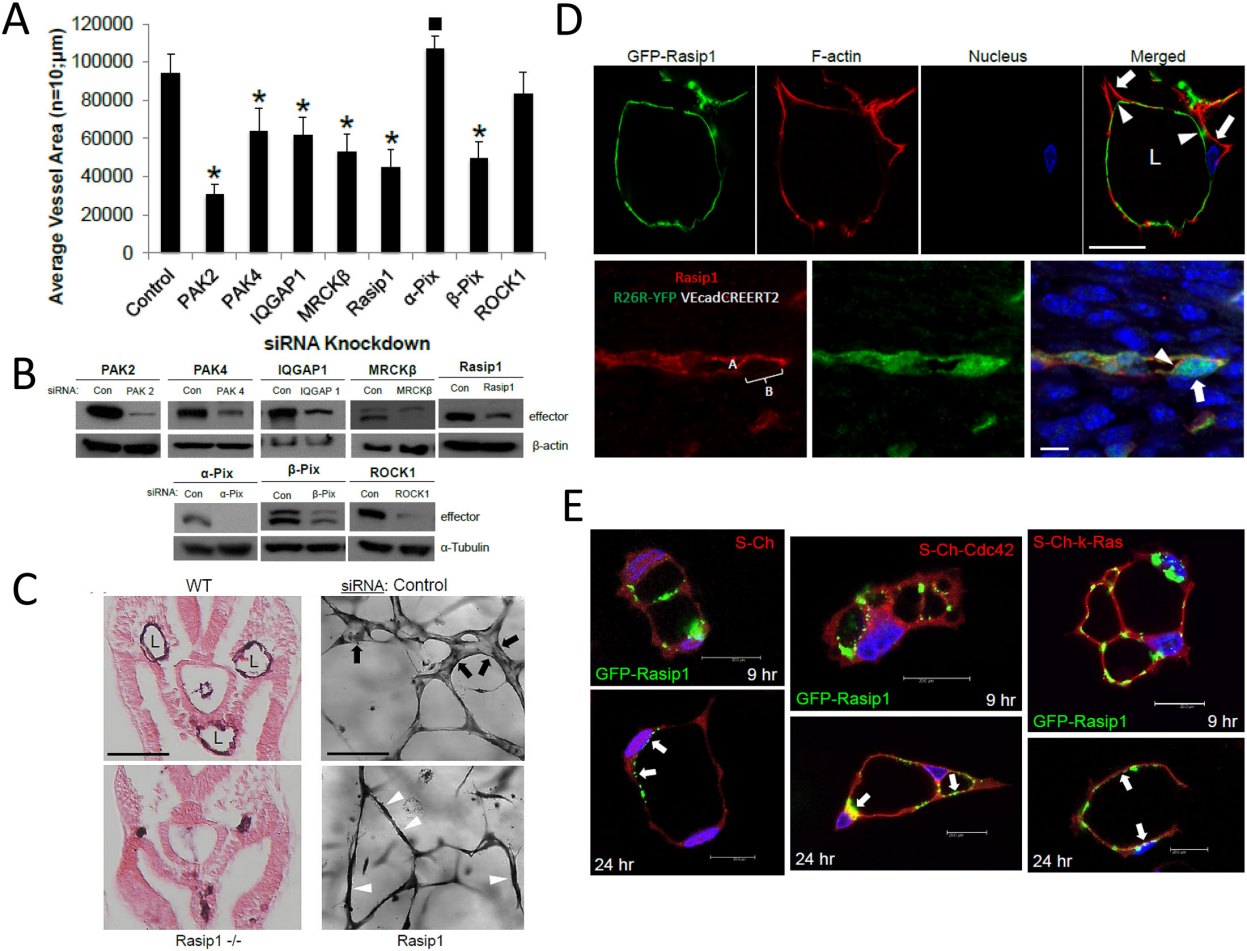
IQGAP1, MRCKβ, Rasip1, and beta-Pix are critical downstream effectors of small GTPase signaling that control EC tubulogenesis: Rasip1 controls EC tubulogenesis in vivo during mouse development and targets to the EC apical surface in vitro and in vivo. (A) Individual EC cultures were transfected with a control siRNA or siRNAs that are directed to the indicated molecules and were then suspended in 3D collagen matrices for 72 hr using the Factor-induced model. Data are reported as mean vessel area ± SD per HPF (n = 10; p < 0.01). Asterisk indicates significance below control while square indicates significance above control. (B) Lysates generated from siRNA transfected cultures in (A) were used in Western Blot analysis and probed for the indicated molecules compared to tubulin controls to assess siRNA suppression. (C) Left panels- Wild type vs. Rasip1 knockout mice were cross-sectioned at E8 and were stained with CD31 antibodies. Vascular lumens (L) are observed in the wild type but not the knockout mice. Bar equals 500 μm. Right panels- ECs were treated with control vs. Rasip1 siRNAs, were seeded in 3D collagen matrices, and after 72 hr, were fixed, stained and photographed. Black arrows indicate EC tubes with lumens, while white arrowheads indicate EC cords without lumens mimicking the *in vivo* observations seen in the left panels. Bar equals 50 μm. (D) Rasip1 is shown to target to the EC apical surface during tubulogenesis *in vitro* vs. *in vivo*. Upper panel- ECs were infected with adenoviruses carrying GFP-Rasip1 and PKCɛ, were cultured in 3D matrices and after 24 hr, cultures were fixed, and stained with phalloidin. Arrowheads indicate apical labeled of Rasip1, while the arrows indicate basal labeling of F-actin. Bar equals 25 μm. Lower panel- Immunofluorescent staining of Rasip1 in developing mouse vessels demonstrates apical targeting of Rasip1. Arrowheads indicate apical staining of Rasip1 (also labeled A), while the arrow indicates a basal region without staining (also labeled B). Bar equals 5 μm. (E) ECs were induced to express GFP-Rasip1, mCherry (Ch), Ch-Cdc42, and Ch-k-Ras, allowed to undergo lumen formation, then fixed at the indicated times and imaged by confocal microscopy. Arrows indicate apical and sub-apical targeting of Rasip1 during different stages of EC lumen formation. Bars equal 20 μm.

Previous work had identified Pak2, Pak4, and Par6b as key effectors of Cdc42 and Rac1-dependent EC lumen formation [8]. Par6 binds Par3 which has affinity for the adhesion molecules, JamB, JamC, and VE-cadherin, which we and others have shown are critical to EC lumen formation and cell polarity during this process [8, 14, 16, 19]. Here, we report the identity of new critical effectors in this lumen formation process and they are IQGAP1 (a Cdc42 and Rac effector and direct regulator of Raf and Erk activation), MRCKβ (a Cdc42 effector and regulator of Cdc42-dependent cell polarization), Rasip1 (a Ras and Rap effector), GIT1 (a Pak2-binding protein), and beta-Pix, a Pak2 binding partner, a known Cdc42 and Rac effector, and a GEF for these GTPases (Fig 6, S2 and S3 Figs). siRNA suppression of each of these effectors showed marked inhibitory effects on the EC lumen and tube formation process. We also compared them to the effects of Pak2 and Pak4 siRNA suppression. No effects were observed from siRNA suppression of alpha-Pix (which shows modest inhibitory activity) and Rock1, a known RhoA effector (Fig 6 and S3 Fig).

To address if we could observe additive or synergistic effects of knockdown of multiple effectors, we performed combination experiments with more than one siRNA (S2 and S3 Figs). We also predicted that this type of analysis might facilitate our ability to delineate if these effectors were in the same or distinct signaling pathways affecting different steps in the EC lumen formation cascade. The greatest blocking combination of siRNAs is when ECs are treated with Pak2 and Pak4, suggesting their critical involvement and their likely participation in different steps of the process because of the strong additivity or possible synergism (S2 and S3 Figs). Interestingly, both Pak2 and Pak4 siRNAs also additively block with siRNAs to MRCKβ, Rasip1 and beta-Pix, while Pak2 more selectively added to the blocking effects of IQGAP1 siRNA (S2 Fig). Additive blocking effects were observed with Rasip1 with Pak2, Pak4, IQGAP1 and MRCKβ siRNAs, but not with beta-Pix, suggesting the possibility that they are functionally linked in a signaling pathway. Additive blocking effects were seen with MRCKβ combined with Pak2, Pak4, Rasip1, but not with either IQGAP1 or beta-Pix. Finally, IQGAP1 siRNA showed additive blocking effects with Pak2 and Rasip1, and beta-Pix siRNA induced additive blocking effects when combined with either Pak2 or Pak4, but not the other effectors. A key point is that these molecules represent critical effectors of lumen formation which are directly linked to Cdc42-, Rac1-, Rac2-, and k-Ras-dependent signaling and when we suppress the expression of these in combination (S2 Fig), there is a profound interference in the ability of ECs to form tubes.

### EC tubulogenesis is controlled by Rasip1 *in vitro* and *in vivo*, and Rasip1 localizes to the EC apical membrane during lumen formation

Previous work demonstrated a role for Rasip1 in EC lumen formation *in vivo* and *in vitro* [11]. Here, we extend these studies to show that using our new Factor system, Rasip1 siRNA treatment reveals a phenotype that directly recapitulates what we observe *in vivo* in the Rasip1 knockout animals (Fig 6). EC cords are observed to form and that reach their appropriate locations in the embryo, but they fail to lumenize (Fig 6C). We observe this exact phenotype *in vitro* in that they effectively assemble into cords in a pattern that resembles that of the control siRNA culture, but they fail to form lumens (Fig 6C). Thus, the *in vivo* knockout data is clearly reflected in the *in vitro* assays which show the same morphogenic phenotype. Our key findings using *in vitro* models of EC lumen formation and EC-pericyte tube co-assembly have repeatedly been demonstrated to be recapitulated in multiple species including Zebrafish, quail and mice.

To assess further a possible role for Rasip1 during these events, we constructed a vector carrying GFP-Rasip1 and transduced it into ECs and performed lumen formation assays. In addition, we increased PKCɛ expression which enhanced targeting of GFP-Rasip1 to the EC apical surface during lumen formation (Fig 6D). We also co-expressed GFP-Rasip1 with other mCherry control or GTPase fusion proteins and in all cases Rasip1 is observed to target apical surfaces during lumen formation over time (Fig 6E). In addition, immunofluorescence staining *in vivo* during mouse vascular development shows a similar distribution of Rasip1 which is observed more selectively along the EC apical surface during initiation of lumen opening (Fig 6D). These data suggest that Rasip1 can target the EC apical surface and this ability correlates with its ability to control lumen formation like the apical/subapical targeting of the key small GTPases (Fig 3 and S1 Fig). Overall, this work shows that membrane transfer events from basal to apical underlie the molecular mechanisms that control the development and expansion of the EC apical membrane surface during lumen and tube formation.

### Real-time video analysis reveals critical involvement of key GTPases, GAPs, and effectors during EC tubulogenesis

Another experimental approach that we have taken here is to perform real-time video analysis of EC tubulogenic responses of control vs. siRNA treated ECs. Control siRNA-treated cells form extensive networks of tubes over the 72 hr time period. During the first 12 hr and later on also, vesicular trafficking and intracellular vacuole formation is observed which contributes to the lumen formation process (S1 Vid) and is demonstrated in our confocal imaging shown earlier (Fig 3). siRNA suppression of Cdc42 markedly interferes with EC tubulogenesis (S2 Vid). Interestingly, it appears that the ECs with reduced Cdc42 are attempting to form lumens and tubes, but then they collapse. In contrast, siRNA suppression of RhoA in ECs allows them to form lumens and tubes just like the control siRNA-treated ECs (S3 Vid). siRNA suppression of Arhgap31 (S4 Vid) and Rasa1 (S5 Vid) which activates Cdc42, Rac1 and k-Ras, respectively, leads to an acceleration of lumen and tube formation. In contrast, siRNA knockdown of Arhgap29, leading to activation of RhoA, leads to inhibition of EC tubulogenesis, where EC cord formation occurs, and attempts at tube formation are observed, but this is followed by collapse of the developing luminal space (S6 Vid). Very similar findings are observed with Rasip siRNA-treated ECs, where EC cord assembly occurs, but lumen formation does not (S7 Vid). Combined siRNA suppression of key lumen regulators including Cdc42 and k-Ras (S8 Vid), Cdc42 and Rac2 (S9 Vid), and Cdc42 and Rap1b (S10 Vid), reveal marked blockade of EC tubulogenesis. The blocking influence of individual siRNAs directed to k-Ras (S11 Vid), Rac2 (S12 Vid), and Rap1b (S13 Vid) are shown as well and finally, these can be directly compared to another field of control siRNA treated ECs which show multicellular EC lumen and tube formation (S14 Vid).

### Differential interactions of small GTPases with downstream effectors as well as MT1-MMP and α2β1 integrin during EC lumen formation

**Fig 7:**
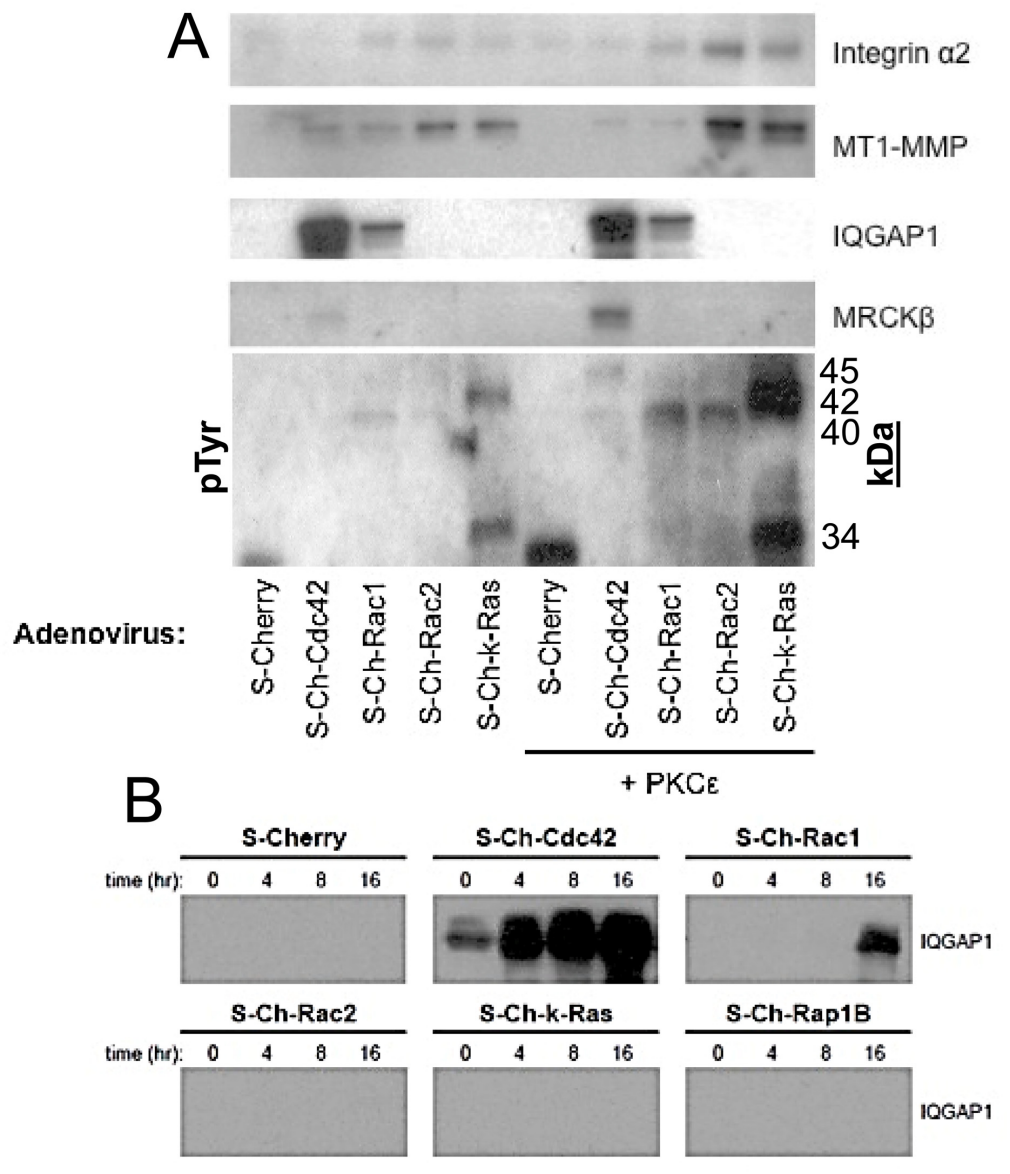
Cdc42, Rac1, Rac2, and k-Ras co-associate with α2 integrin, MT1-MMP and key GTPase effectors in lumen signaling complexes during EC tubulogenesis in 3D collagen matrices. (A) ECs were induced to express S-Cherry, S-Ch-Cdc42, S-Ch-Rac1, S-Ch-Rac2, S-Ch-k-Ras, and with or without PKCɛ. Cultures were then suspended in 3D collagen matrices for 16 hours using the phorbol ester-induced model, detergent lysates from these cultures were then prepared and incubated with S-protein agarose to selectively bind S-epitope tagged proteins and their associated proteins. Eluates were evaluated using Western blot analysis and probed for expression of α2 integrin, MT1-MMP, IQGAP1, MRCKβ, and tyrosine phosphorylated proteins. (B) EC cultures were induced to express S-Cherry, S-Ch-Cdc42, S-Ch-Rac1, S-Ch-Rac2, S-Ch-k-Ras, and S-Ch-Rap1B. Cultures were then suspended in 3D collagen matrices and detergent lysates were prepared at 0, 4, 8, and 16 hr. Lysates were incubated with S-protein agarose and eluates were examined for expression of IQGAP1.

To further address the functional biochemical connections of the GTPases and effectors in the EC lumen formation cascade, we performed pull-down assays using our S-Cherry GTPases constructs during this process in 3D matrices. We also stimulated lumen formation by increasing the expression of PKCɛ in order to assess if the pulldown assays correlate with the functional enhancement of lumen formation (Fig 7) (see Fig 2C). Consistently in our pulldown experiments, S-Ch-Rac2 and S-Ch-k-Ras co-precipitate with MT1-MMP, but not S-Cherry control while lesser but detectable interactions were also observed with Cdc42 and Rac1. These pulldowns are enhanced by increased expression of PKCɛ. Similar results are observed with α2β1 pulldown and this is consistent with our previous observations demonstrating an interaction between MT1-MMP and α2β1 integrin [14]. This demonstrates that two key regulators of lumen formation, MT1-MMP and α2β1, interact in multiprotein complexes with key small GTPases controlling lumen formation, namely, Rac2, k-Ras, Rac1 and Cdc42, and this is stimulated by increased PKCɛ expression.

IQGAP1 strongly interacts with Cdc42 and also to a lesser extent with Rac1 (Fig 7A), but not the other proteins and these interactions increase as lumen formation proceeds (Fig 7B). MRCKβ interacts selectively with Cdc42 during the lumen formation process and this interaction is enhanced by increased PKCɛ expression (Fig 7A). Interestingly, MRCKβ is activated by diacylglycerol, like PKCɛ. Finally, we performed these experiments and blotted for phosphotyrosine-containing proteins and identified unique interactions for the different GTPases (Fig 7). k-Ras interacts with two phosphotyrosine-containing proteins at 42 and 34 kDa, while Rac1 and Rac2 interact with a band at 40 kDa and Cdc42 binds to a 45 kDa band (Fig 7A). The identity of each of these bands will be pursued in future studies. When PKCɛ expression is enhanced, each of these bands increases their respective GTPase association. These data indicate unique associations of each GTPase during EC lumen formation.

## Discussion

A critical question in vascular biology is how ECs assemble networks of tubes with defined lumens [2–5]. A primary function of ECs is to undergo tubulogenesis, maintain tube structures, and then specialize into ECs with unique functions tailored to meet specific tissue requirements. Here, we focused on the molecular mechanisms underlying EC lumen and tube assembly. We identify new GTPase regulators of EC lumen formation as well as downstream regulators of these GTPases. In addition to reaffirming a key role for Cdc42, Rac1 and their effectors, Pak2 and Pak4, we demonstrate novel roles for Rac2, k-Ras, Rap1b, and the effectors, IQGAP1, MRCKβ, beta-Pix, GIT1 and Rasip1. Furthermore, we demonstrate important new roles for three GAPs: Arhgap31 which inactivates Cdc42 and Rac, Rasa1 which inactivates k-Ras, and Arhgap29 which inactivates RhoA. Arhgap31 and Rasa1 siRNAs were shown individually and in combination to stimulate tube formation (via blockade of Cdc42, Rac, and Ras respectively), while Arhgap29 siRNA inhibits tube formation (via blockade of RhoA). The above molecules appear to control EC tubulogenesis by affecting asymmetric cytoskeletal polarization (i.e. modified tubulins expressed subapically, F-actin basally) and signal transduction events which are necessary to direct membrane trafficking toward the new apical surface to create a defined lumen in a 3D environment. Furthermore, we demonstrate that Cdc42, Rac, k-Ras, Rasip1, and phospho-c-Raf accumulate at the developing apical membrane, while acetylated tubulin is strongly localized subapically to support this apical surface. Interestingly, in focal regions within this subapical domain, there is strong co-localization of Cdc42 with acetylated tubulin.

**Fig 8:**
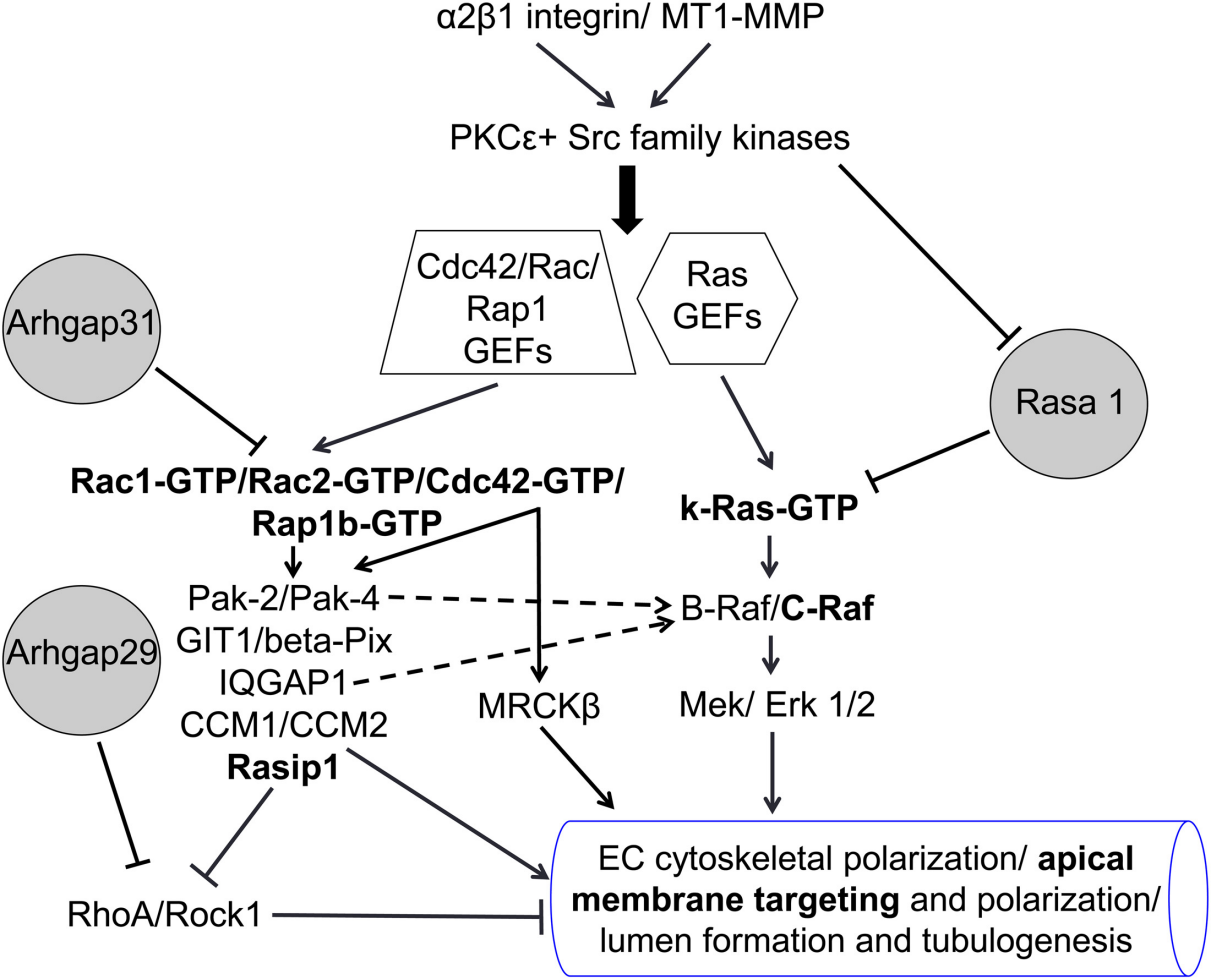
EC tubulogenesis in 3D collagen matrices is controlled by activation of a Cdc42-, Rac-, and k-Ras-dependent signaling cascade: A process that is antagonized by Arhgap31, Rasa1 and RhoA. A schematic diagram is shown illustrating key molecules and signals that control how ECs form lumens and tubes in 3D collagen matrices. These molecules and signals regulate EC cytoskeletal polarization (acetylated and detyrosinated tubulin- subapical; F-actin- basal), and the generation of the apical membrane which is decorated with key small GTPases controlling these processes including Cdc42, Rac1, Rac2, k-Ras and Rap1b, and the effectors, c-Raf and Rasip1 (highlighted in bold). EC tubulogenesis also requires MT1-MMP-dependent matrix proteolysis, a step that is co-dependent and coordinated with the indicated GTPase-, effector-, integrin- and kinase-dependent signaling cascades. Intracellular vacuoles and vesicles (strongly labeled with Rac1 and k-Ras) traffic along subapically oriented acetylated tubulin tracks, and then fuse together in a polarized perinuclear region (where acetylated tubulin co-localizes with Cdc42) to generate and expand the EC apical surface.

A key conclusion of our findings is that small GTPase-mediated signaling in conjunction with a kinase signaling cascade involving PKCɛ, Src, Pak, Raf and Erk kinases leads to four major events that control EC tubulogenesis (Fig 8). The first is the establishment of asymmetric cytoskeletal polarity with subapical modified tubulins (particularly acetylated tubulin, but also detyrosinated tubulin), and F-actin distributed in a strong basal location. Our work suggests that this is necessary to direct membrane trafficking along subapically polarized microtubule tracks which appears to facilitate focal apical accumulation of vesicles/ vacuoles and subsequent vesicle fusion events to create the luminal space. The membranes that become the apical membrane surface are clearly enriched in small GTPases that affect EC lumen and tube formation including those that we evaluate here: Cdc42, Rac1, Rac2, k-Ras, and Rap1b. The second process is the creation of a polarized apical membrane surface. Vesicle/vacuole trafficking to this apical membrane appears to occur along the microtubule cytoskeleton and they accumulate in a polarized, perinuclear region. Thirdly, vesicle-to-vesicle fusion events (from vesicles derived from pinocytosed/ endocytosed membranes or other intracellular membranes derived from endoplasmic reticulum, Golgi or Weibel-Palade bodies) occur to generate an apical membrane template, which can then be further remodeled over time through endocytic and exocytic membrane trafficking events to develop a mature EC apical membrane domain. The molecular composition of this EC apical membrane domain is not well understood and ongoing work is addressing how it is assembled, remodeled, and maintained. Published real-time videos from our laboratory have revealed the dynamic nature of these membrane trafficking and vesicle fusion events which occur rapidly over a 4–72 hr period to create networks of capillary tubes with defined lumens [2, 10, 11, 26, 38]. The fourth process that is coordinated with the other three events is cell surface proteolysis through MT1-MMP, which is required to create matrix-free spaces (termed vascular guidance tunnels) that are necessary for lumens and tube networks to form [10]. Vascular guidance tunnels form during EC tubulogenesis in either collagen or fibrin matrices to serve as matrix-free conduits in 3D matrices that affect EC tube formation, remodeling, and EC-pericyte interactions leading to vascular basement membrane matrix assembly [10, 22, 39].

Our new work demonstrates that multiple small GTPases contribute to EC tube formation, and we chose to focus our attention on Cdc42, Rac1, Rac2, k-Ras and Rap1b. siRNA suppression of Cdc42 in combination with k-Ras, Rac2 and Rap1b resulted in the most profound blocking effects, while blockade of Pak2 (activated by both Cdc42 and Rac isoforms) combined with multiple other effectors resulted in similar very strong blocking effects. Similar marked blocking effects were observed with Rasip1 siRNA suppression combined with either IQGAP1 or MRCKβ, which are Ras, Rac and Cdc42 effectors [40–43]. This is highly supportive again of the conclusion that multiple GTPases and effectors work in concert to control this process. This is also demonstrated by siRNA suppression of Arhgap31 and Rasa1 together (to stimulate Cdc42, Rac and Ras activity in combination), where marked stimulation of EC tube formation occurs. Interestingly, other work supports our general conclusions, in that IQGAP1 is known to directly interact directly with k-Ras, b-Raf, and Erk [40, 44, 45]; thus serving as a scaffolding protein to promote Erk activity downstream of Cdc42, Rac, and Ras activation. In addition, Cdc42 and MRCKβ have been implicated in nuclear positioning in conjunction with centrosome reorientation during cell motility events [46], and this process may also be critical during EC lumen formation since we observe accumulation of vesicles/vacuoles in a polarized perinuclear region. Src activity has been reported to inhibit the Rho/Rock pathway [47], a pathway also inhibited by Pak2/Pak4 [48], Rasip1/Arhgap29 [11], and the cerebral cavernous malformation (CCM) proteins, CCM1 and CCM2 [15, 17]. Interestingly, Rap1 is known to affect the function of Rasip1/Arhgap29 [49], and CCM1 is an effector for Rap1 [50]. In addition, Src activation blocks the activity of Rasa1 [51], while stimulating Pak2/Pak4 activation and Raf activation [9]. Interestingly, the Cdc42 guanine exchange factor (GEF), intersectin1, has been reported to inhibit the activity of Arhgap31 [52]. Future work in our laboratory will focus on the identification of the relevant GEFs which activate the key small GTPases that we have identified which control EC lumen and tube formation.

A major regulator of EC tubulogenesis is membrane trafficking from basal to apical, but possibly also from other intracellular membranes to the apical surface. In one of our first studies we demonstrated the presence of von Willebrand factor present inside intracellular vacuoles suggesting potential fusion of Weibel-Palade bodies with these vacuoles [23]. We observe the presence of intracellular vacuoles which are macropinosome-like structures (the vacuoles are strongly labeled by including membrane impermeant fluorescent dyes into the culture media showing that they are pinocytic) that are transported toward the apical surface to fuse with the luminal membrane [23]. These vacuole membranes can clearly be shown to possess Rac1 (very strongly observed when constitutively active Rac is utilized) suggesting that active Rac targets these membranes. Cdc42 appears to surround these structures *in vitro* and *in vivo* (when GFP-Cdc42 was expressed in ECs in Zebrafish embryos) [7, 26, 53] and can be observed to accumulate subapically along with acetylated tubulin where focal areas of strong co-localization can be seen. These are also regions of probable vacuole-vacuole and vacuole-apical membrane fusion events that control the creation of the polarized luminal surface. Our new work suggests that Rasip1 can target apically and this is observed to a greater extent when we increase the expression of PKCɛ, a stimulus that markedly accelerates EC lumen and tube formation. Past work suggests that Ras can interact with Rasip1 [43], so there may be a direct relationship with these findings, more studies will need to investigate these potential connections. Also, further work will need to address the role of different membrane compartments (basal to apical transfer through intracellular vacuoles) or other intracellular membranes (vesicle trafficking from endoplasmic reticulum or Golgi, or trafficking from structures such as Weibel-Palade bodies), and determine how individual proteins such as Cdc42, k-Ras, Rasip1 and Rac1 target to the EC apical domain and influence this process.

One of the intriguing questions in cell biology is what are the necessary molecules and signals that are required for cells to generate lumens and tubes and what distinguishes them from cells that cannot. ECs and many types of epithelial cells can form tube structures, while cell types such as fibroblasts, pericytes, and vascular smooth muscle cells are unable to do so. Of great interest is that we have identified a key series of molecules and signals that are necessary for ECs to form lumens and tubes. Genetic or chemical blockade of these molecules and signals converts ECs into cells that lose this ability to form lumens. We also presented data showing inhibitory roles for the small GTPase GAPs, Rasa1 and Arhgap31, and perhaps the lumen formation signaling cascade suppresses their activity. Furthermore, previous work has suggested that EC lumen signaling suppresses RhoA- and Rock-dependent signaling and key EC molecules such as Rasip1, Arhgap29, CCM proteins, Src, Pak2 and Pak4 are known to suppress Rho/Rock activation [3, 4]. In addition, our previous work has identified other inhibitors of EC lumen formation including HDAC6, Sirt2, Csk, TIMP2 and TIMP3 [9, 29, 38]. It is becoming increasing clear that EC tube formation is controlled by a balance of stimulatory and inhibitory molecules (Fig 8). Vessel abnormalities could result from imbalances of signals from either one of these sets of molecules. How such a balance controls the ability of ECs to form tubes and other cells such as fibroblasts to not form tubes is a key cell biological problem and question that needs to be investigated in detail in future studies.

In addition to our efforts to elucidate how ECs form lumens and tubes in 3D matrices, we have previously investigated another key way in which ECs within capillaries become polarized and that is the selective recruitment of pericytes to the EC abluminal surface [20, 28, 39]. Of great interest, pericyte recruitment to EC-lined tubes in capillaries leads to another critical event which is the abluminal and, thus, polarized deposition of the vascular basement membrane, a process that requires both cell types [28, 39, 54]. An important question for future studies is how the tubulogenic regulators that we describe impact the ability of EC cord and tube networks to attract pericytes and induce vascular basement membrane assembly. Thus, the tubulogenic mechanism that we describe here is an essential and fundamental step in EC polarity and vessel maturation which is further amplified by the recruitment and retention of pericytes around capillary tubes.

## Supporting Information

S1 Fig Apical Polarization of small GTPases and effectors that control EC tubulogenesis. ECs were induced to express Cherry control, the indicated Cherry-GTPases or GFP-Cdc42 fusion proteins and lumen formation assays were performed for 24 hrs. (A,B) Confocal images reveal apical targeting of the indicated GTPases (arrowheads), and arrows indicate basal targeting of F-actin. Bar equals 25 μm. (C) Apical targeting of activated c-Raf during EC lumen formation (arrows) compared to basal targeting of F-actin (arrowheads). Bar equals 50 μm. (D) Increased expression of Cdc42, k-Ras and Rap1b leads to accelerated lumen formation. (E) The HDAC6 inhibitor, tubacin, stimulates EC lumen formation. Asterisks indicates significance at p < .01 compared to control (n = 12).
(TIF)

S2 Fig Cdc42- and Rac-dependent signaling affect tubulin modifications that control EC lumen formation and apical polarization: Key role of small GTPase effectors during EC tubulogenesis. (A) Left panel- ECs were induced to express the indicated dominant negative mutants and lumen cultures were lysed and probed with acetylated tubulin vs. control antibodies. Right panel- ECs were induced to express the indicated wild-type proteins or constitutively active Rac1 mutant and lumen cultures were lysed and probed with acetylated tubulin, detyrosinated tubulin or control antibodies. (B) ECs were treated with the indicated siRNAs, singly or in combinations of two, and EC lumen assays were performed, fixed, photographed and quantitated. Squares indicate significance at p < .01 compared to control, while asterisks indicate significance at p < .01 compared to the indicated single siRNA (n = 10). (C) siRNA suppression of GIT1 reveals a role in EC tubulogenesis. Asterisk indicates significance at p < .01 compared to control. Bar equals 100 μm.
(TIF)

S3 Fig Small GTPase effectors are fundamental regulators of EC tubulogenesis in 3D matrices. ECs were treated with the indicated siRNAs singly (A) or in combination (B) and tube forming assays were performed and fixed after 72 hr. Representative photographs of the cultures are shown. Bar equals 25 μm for A and 50 μm for B.
(TIF)

S1 Video ECs were treated with control siRNA and were allowed to form networks of tubes in 3D collagen matrices over 72 hr and in response to the Factors under serum-free media conditions. Considerable movement of the ECs is observed, with intracellular vacuolation and membrane trafficking events controlling the lumen formation process. ECs assemble together through motility towards each other leading to multicellular EC tubes with defined lumens. The video is shown at 7.7 frames/sec.
(MOV)

S2 Video ECs were treated with a Cdc42 siRNA and were suspended in 3D collagen over 72 hr and in response to the Factors under serum-free media conditions. ECs show motility and move towards each other leading to some cell-cell clusters, but they fail to form sustained lumens and tubes. There are attempts by the cells to form lumen structures but they are unable to sustain them. The video is shown at 7.7 frames/sec.
(MOV)

S3 Video ECs were treated with a RhoA siRNA and were suspended in 3D collagen over 72 hr and in response to the Factors under serum-free media conditions. The treated ECs are readily observed to form lumen and tube structures in a manner similar to control siRNA-treated ECs. The video is shown at 7.7 frames/sec.
(MOV)

S4 Video ECs were treated with an Arhgap31 siRNA and were suspended in 3D collagen over 72 hr and in response to the Factors under serum-free media conditions. The treated ECs are readily observed to form lumen and tube structures in an accelerated manner due to increased Cdc42 and Rac activity compared to control siRNA-treated ECs. The video is shown at 7.7 frames/sec.
(MOV)

S5 Video ECs were treated with a Rasa1 siRNA and were suspended in 3D collagen over 72 hr and in response to the Factors under serum-free media conditions. The treated ECs are readily observed to form lumen and tube structures in a markedly accelerated manner due to increased Ras activity compared to control siRNA-treated ECs. The video is shown at 7.7 frames/sec.
(MOV)

S6 Video ECs were treated with an Arhgap29 siRNA and were suspended in 3D collagen over 72 hr and in response to the Factors under serum-free media conditions. The treated ECs show the ability to form cords and co-assemble, but attempts at lumen and tube formation are rapidly followed by apparent tube collapse secondary to increased RhoA activity compared to control siRNA-treated ECs. The video is shown at 7.7 frames/sec.
(MOV)

S7 Video ECs were treated with a Rasip1 siRNA and were suspended in 3D collagen over 72 hr and in response to the Factors under serum-free media conditions. The treated ECs show a strong ability to form cords, like we see in in vivo mouse knockout animals, and co-assemble with other ECs. Apparent attempts at lumen formation are rapidly collapsed leaving only networks of cords with no defined lumen space. The video is shown at 7.7 frames/sec.
(MOV)

S8 Video ECs were treated with both Cdc42 and k-Ras siRNAs and were suspended in 3D collagen over 72 hr and in response to the Factors under serum-free media conditions. ECs show reduced motility and some EC-EC interactions, but no lumen and tube formation, so this combination of siRNAs markedly blocks these processes. The video is shown at 7.7 frames/sec.
(MOV)

S9 Video ECs were treated with both Cdc42 and Rac2 siRNAs and were suspended in 3D collagen over 72 hr and in response to the Factors under serum-free media conditions. ECs show motility, some EC-EC interactions, some interacting cells are seen to come apart, but overall, there is no lumen and tube formation observed. The video is shown at 7.7 frames/sec.
(MOV)

S10 Video ECs were treated with both Cdc42 and Rap1b siRNAs and were suspended in 3D collagen over 72 hr and in response to the Factors under serum-free media conditions. ECs show motility, reduced EC-EC interactions, and interacting ECs appear to disassemble are seen to come apart, but overall, there is no lumen and tube formation observed. The video is shown at 7.7 frames/sec.
(MOV)

S11 Video ECs were treated with a k-Ras siRNA and were suspended in 3D collagen over 72 hr and in response to the Factors under serum-free media conditions. ECs show motility, EC-EC interactions, and attempts at lumen and tube formation, but overall, the transient structures that are formed are not sustained. The video is shown at 7.7 frames/sec.
(MOV)

S12 Video ECs were treated with a Rac2 siRNA and were suspended in 3D collagen over 72 hr and in response to the Factors under serum-free media conditions. ECs show motility, prominent EC filopodia, EC-EC interactions, and attempts at lumen and tube formation, but overall, EC lumen and tube formation is markedly inhibited. The video is shown at 7.7 frames/sec.
(MOV)

S13 Video ECs were treated with a Rap1b siRNA and were suspended in 3D collagen over 72 hr and in response to the Factors under serum-free media conditions. ECs show motility, EC-EC interactions with networks of cords, but lumen and tube formation does is strongly inhibited. The video is shown at 7.7 frames/sec.
(MOV)

S14 Video ECs were treated with control siRNA and were allowed to form networks of tubes in 3D collagen matrices over 72 hr and in response to the Factors under serum-free media conditions. Motility of the ECs is observed, with intracellular vacuolation and membrane trafficking events controlling the lumen formation and tube assembly process. The video is shown at 7.7 frames/sec.
(MOV)
