# Supplementary figures and images for: Cdc42 and k-Ras Control Endothelial Tubulogenesis through Apical Membrane and Cytoskeletal Polarization: Novel Stimulatory Roles for GTPase Effectors, the Small GTPases, Rac2 and Rap1b, and Inhibitory Influence of Arhgap31 and Rasa1
Source: PLoS One. 2016 Jan 26;11(1):e0147758. doi: 10.1371/journal.pone.0147758 (PMC4728208; doi:10.1371/journal.pone.0147758)

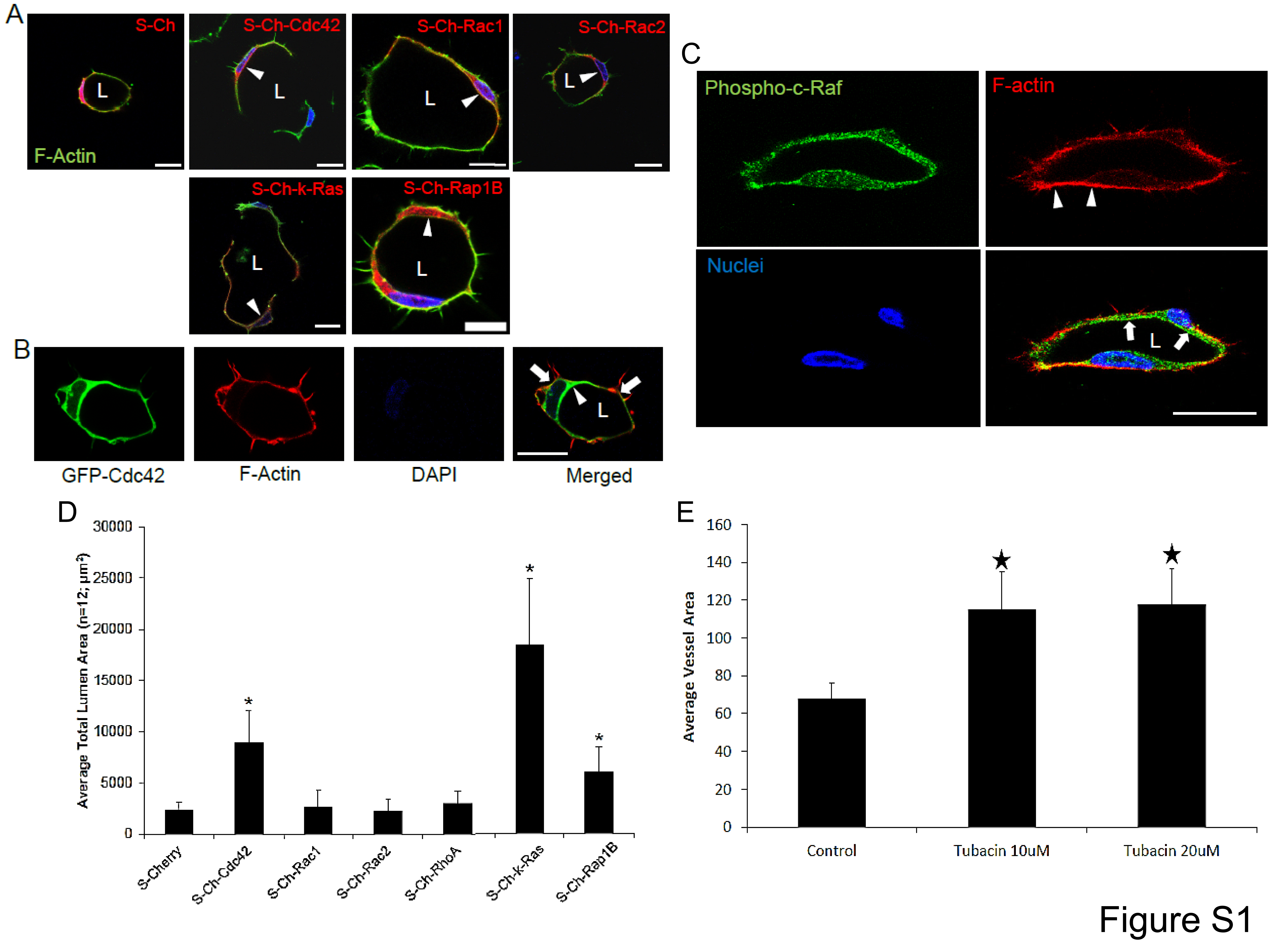

Supplement: S1 Fig — ECs were induced to express Cherry control, the indicated Cherry-GTPases or GFP-Cdc42 fusion proteins and lumen formation assays were performed for 24 hrs. (A,B) Confocal images reveal apical targeting of the indicated GTPases (arrowheads), and arrows indicate basal targeting of F-actin. Bar equals 25 μm. (C) Apical targeting of activated c-Raf during EC lumen formation (arrows) compared to basal targeting of F-actin (arrowheads). Bar equals 50 μm. (D) Increased expression of Cdc42, k-Ras and Rap1b leads to accelerated lumen formation. (E) The HDAC6 inhibitor, tubacin, stimulates EC lumen formation. Asterisks indicates significance at p < .01 compared to control (n = 12). (TIF) [file pone.0147758.s001.tif]

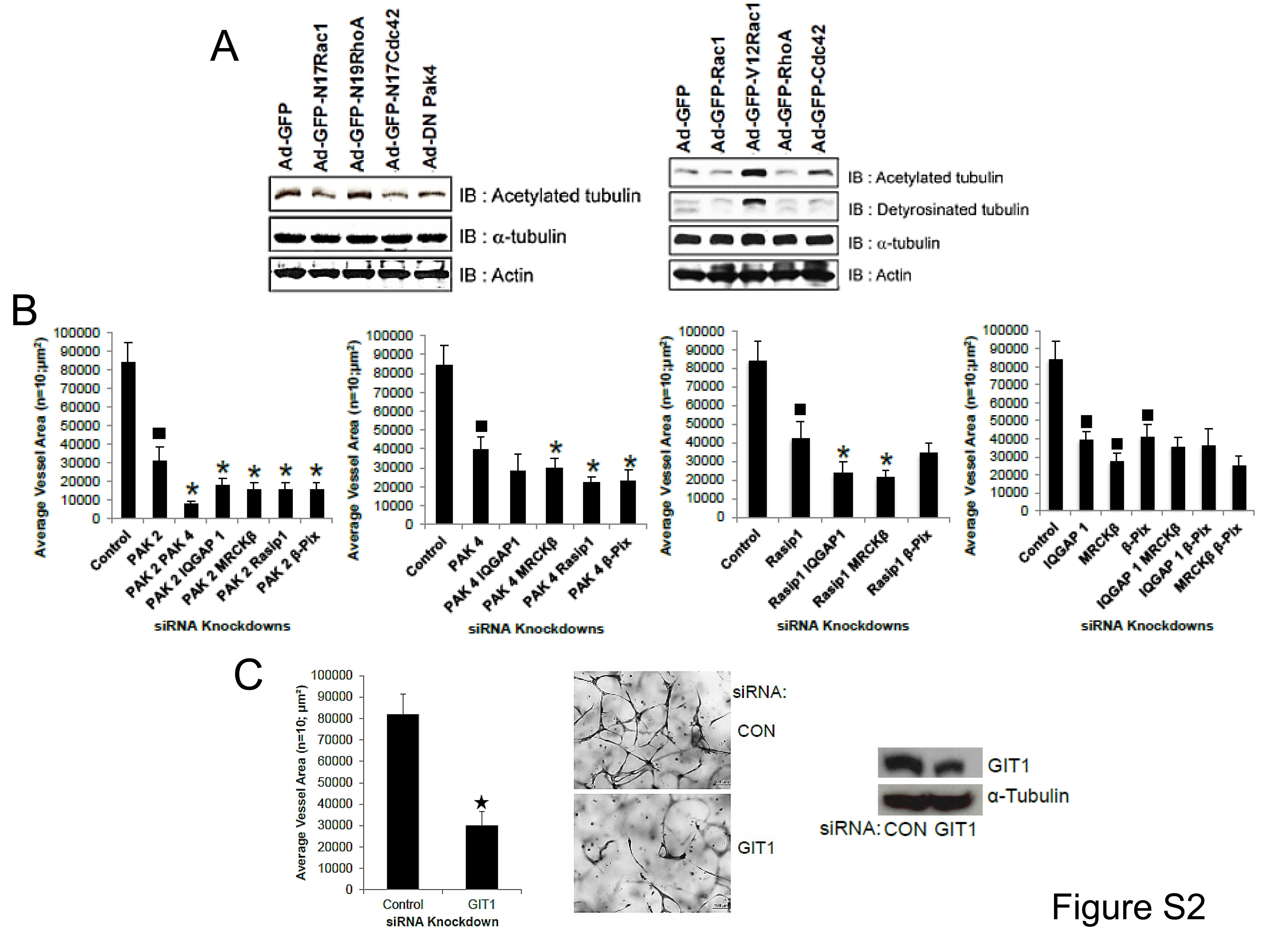

Supplement: S2 Fig — (A) Left panel- ECs were induced to express the indicated dominant negative mutants and lumen cultures were lysed and probed with acetylated tubulin vs. control antibodies. Right panel- ECs were induced to express the indicated wild-type proteins or constitutively active Rac1 mutant and lumen cultures were lysed and probed with acetylated tubulin, detyrosinated tubulin or control antibodies. (B) ECs were treated with the indicated siRNAs, singly or in combinations of two, and EC lumen assays were performed, fixed, photographed and quantitated. Squares indicate significance at p < .01 compared to control, while asterisks indicate significance at p < .01 compared to the indicated single siRNA (n = 10). (C) siRNA suppression of GIT1 reveals a role in EC tubulogenesis. Asterisk indicates significance at p < .01 compared to control. Bar equals 100 μm. (TIF) [file pone.0147758.s002.tif]

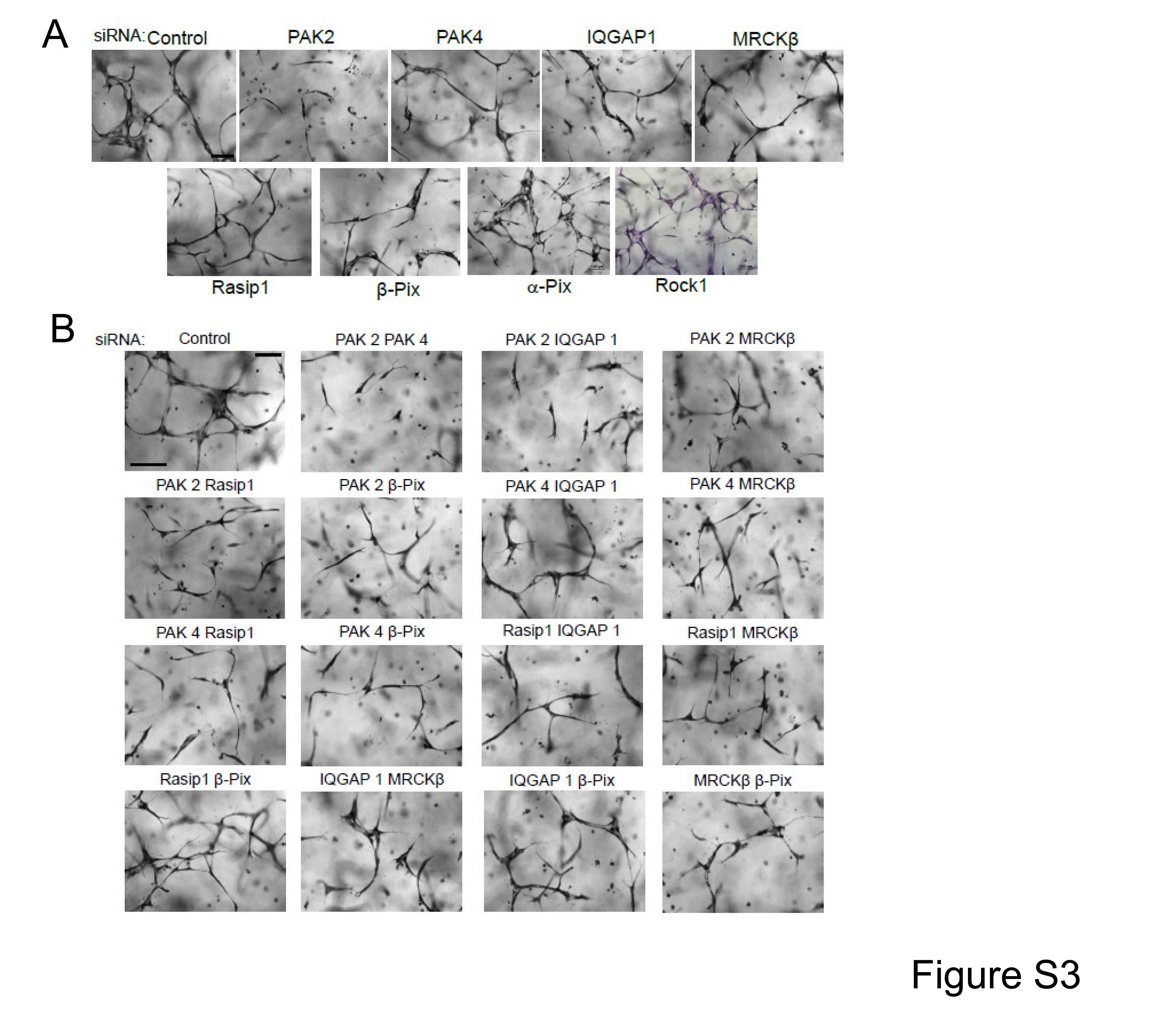

Supplement: S3 Fig — ECs were treated with the indicated siRNAs singly (A) or in combination (B) and tube forming assays were performed and fixed after 72 hr. Representative photographs of the cultures are shown. Bar equals 25 μm for A and 50 μm for B. (TIF) [file pone.0147758.s003.tif]
